# Multiple gene-specific DNA methylation in blood leukocytes and colorectal cancer risk: a case-control study in China

**DOI:** 10.18632/oncotarget.18054

**Published:** 2017-05-22

**Authors:** Yupeng Liu, Yibaina Wang, Fulan Hu, Hongru Sun, Zuoming Zhang, Xuan Wang, Xiang Luo, Lin Zhu, Rong Huang, Yan Li, Guangxiao Li, Xia Li, Shangqun Lin, Fan Wang, Yanhong Liu, Jiesheng Rong, Huiping Yuan, Yashuang Zhao

**Affiliations:** ^1^ Department of Epidemiology, Public Health College, Harbin Medical University, Harbin 150081, Heilongjiang Province, The People’s Republic of China; ^2^ Department of Clinical Laboratory, The Second Affiliated Hospital of Harbin Medical University, Harbin 150081, Heilongjiang Province, The People’s Republic of China; ^3^ Department of Orthopedics Surgery, The Second Affiliated Hospital of Harbin Medical University, Harbin 150081, Heilongjiang Province, The People’s Republic of China; ^4^ Key Laboratory of Ophthalmology, Department of Ophthalmology, The Second Affiliated Hospital of Harbin Medical University, Harbin 150081, Heilongjiang Province, The People’s Republic of China

**Keywords:** colorectal cancer risk, DNA methylation, leukocytes

## Abstract

The relationship between gene-specific DNA methylation in peripheral blood leukocytes and colorectal cancer (CRC) susceptibility is unclear. In this case-control study, the methylation status of a panel of 10 CRC-related genes in 428 CRC cases and 428 cancer-free controls were detected with methylation-sensitive high-resolution melting analysis. We calculated a weighted methylation risk score (MRS) that comprehensively combined the methylation status of the panel of 10 genes and found that the MRS_10 was significantly associated with CRC risk. Compared with MRS-Low group, MRS-High group and MRS-Medium group exhibited a 6.51-fold (95% CI, 3.77-11.27) and 3.85-fold (95% CI, 2.72-5.45) increased risk of CRC, respectively. Moreover, the CRC risk increased with increasing MRS_10 (*P*_trend_ < 0.0001). Stratified analyses demonstrated that the significant association retained in both men and women, younger and older, and normal weight or underweight and overweight or obese subjects. The area under the receiver operating characteristic curves for the MRS_10 model was 69.04% (95% CI, 65.57-72.66%) and the combined EF and MRS_10 model yielded an AUC of 79.12% (95% CI, 76.22-82.15%). Together, the panel of 10 gene-specific DNA methylation in leukocytes was strongly associated with the risk of CRC and might be a useful marker of susceptibility for CRC.

## INTRODUCTION

Colorectal cancer (CRC), with an estimated 1,360,602 newly diagnosed cases and 693,933 deaths in 2012, is the third-most common cancer in men and the second-most in women worldwide [1]. In China, CRC is the fifth-most common cancer in men and the fourth-most in women, with an estimated 376,300 newly diagnosed cases and 191,000 deaths in 2015 [2]. The cancerization of colon mucosal epithelial cells is a complex and multifactorial, gradual process, characterized by the accumulation of cancer-specific genetic and epigenetic alterations. Epigenetic DNA modifications, including aberrant DNA methylation, are recognized as major and causal epigenetic events that occur during CRC initiation [3]. Accumulating evidence suggests that the global hypomethylation of DNA might induce chromosomal instability and gene-specific hypermethylation can silence tumor suppressor genes, all of which might contribute to CRC formation.

Until now, studies have mainly focused on tumor-derived DNA methylation changes, and studied the relationship between DNA methylation status in tumor tissue and prognosis of various cancers including CRC. The aberrant methylated genes in tumor tissue are commonly involved in aspects of cell function such as cell cycle regulation (*CDKN2A* (also known as *p16*) and IGF2), DNA mismatch repair (*MLH1 and MGMT*), apoptosis (*DAPK1*), cell adhesion (*CDH1*) and signal transduction (*APC* and *WIF1*) in CRC [4–10]. It is clear that tumors do not develop as an isolated phenomenon in their target tissue, other organ systems including the immune system (such as peripheral blood leukocytes) are also involved in tumor initiation [11]. In addition, altered DNA methylation in peripheral blood leukocytes is associated with environmental exposures encountered throughout life [12, 13]. Meanwhile, several experiments *in vitro* showed that environmental factors may disrupt epigenetic balance and may cause methylation abnormalities, and these aberrations have been associated with cancer susceptibility [14, 15]. Therefore, DNA methylation alterations in leukocytes may reflect epigenetic modifications, environmental exposures, or interactions between these factors that increase disease susceptibility. However, only a few studies have assessed the association between DNA methylation in leukocytes and the risk of CRC or colorectal adenomas. Some studies focused on genomic methylation of leukocyte DNA in relation to the risk CRC [16–19] or colorectal adenomas [20, 21], and other studies have reported an association between gene-specific methylation in leukocytes and the risk of CRC [22–26] or colorectal adenomas [27–29]. In addition, recently, several studies have reported gene-specific methylation alterations in leukocytes from patients with various cancers, including bladder, breast, renal, and head and neck cancer [30–34]. The results of these studies did imply aberrant methylation of multiple genes in leukocytes might predispose toward susceptibility to CRC, like genetic variants of germline DNA. Therefore, we carried out this case-control study to investigate the associations between leukocyte-derived DNA methylation in a panel of 10 genes and the risk of CRC.

## RESULTS

### Main characteristics of the participants

Table 1 shows the basic characteristics of the cases and their matched controls. The CRC cases exhibited an overall lower body mass index than the controls. Of the 914 eligible DNA samples, methylation status of the 10 genes was successfully assessed in 856 samples (93.65%), which were included in the final analysis. The characteristics of demographic variables and questionnaire-derived variables before and after multiple imputations are listed in Supplementary Table 1.

**Table 1:** Main characteristics of colorectal cancer cases and controls

Characteristics		Number of cases (%)	Number of controls (%)	*P*
Total number		428	428	
Age	Mean (SD)	59.37 (10.30)	59.36 (10.35)	0.99
Gender	Male	266 (62.15)	266 (62.15)	1.00
	Female	162 (37.85)	162 (37.85)	
BMI	<18.50	28 (6.54)	26 (6.07)	<0.0001
	18.5-24.00	219 (51.17)	146 (34.11)	
	24.0-28.00	150 (35.05)	139 (32.48)	
	≥28.00	31 (7.24)	117(27.34)	
Tumour site	Colon	177 (41.35)	-	-
	Rectum	251 (58.65)	-	-
Pathological morphology	Protruding type	237 (55.37)	-	-
	Ulcerative type	135 (31.54)	-	-
	Other types	56 (13.09)	-	-
Degree of differentiation	Low	43 (10.05)	-	-
	Medium	350 (81.78)	-	-
	High	34 (7.94)	-	-
	Unknown	1 (0.23)	-	-
Histological classification	Adenocarcinoma	395 (92.29)	-	-
	Other types	33 (7.71)	-	-
Dukes stage	A-B	231 (53.97)	-	-
	C-D	157 (36.68)	-	-
	Unknown	40 (9.35)	-	-

BMI: body mass index; SD: standard deviation.

### DNA methylation and CRC risk

CRC risk was significantly associated with the methylation of *DAPK1*, *IGF2*, *MINT31*, *NEUROG1* and *WIF1* (Table 2). A marginally significant association was observed for *MGMT*. For the methylation of *APC*, *CDH1*, *p16* and *MLH1*, there were no significant differences between the cases and the controls regardless of adjustment. For MRS_10, compared with the subjects in the MRS-Low group (57.36% of participants), the subjects in MRS-High (11.80% of participants) and MRS-Medium groups (30.84% of participants) exhibited a 6.51-fold (95% CI, 3.77 to 11.27, *P*<0.0001) and 3.85-fold (95% CI, 2.72 to 5.45, *P*<0.0001) increased risk for CRC, respectively. Moreover, the CRC risk increased with increasing MRS_10 (*P_trend_* < 0.0001).

**Table 2:** Associations between methylation at individual genes, MRS_10 and the risk of CRC

DNA methylation status^a^		Cases (%)^b^	Controls (%)^b^	Crude OR	95% CI	*P*	OR_adjusted_^c^	95% CI	*P*	OR_adjusted_^d^	95% CI	*P*
*APC*	Negative	412 (96.26)	419 (97.90)	1.00			1.00			1.00		
	Positive	16 (3.74)	9 (2.10)	1.81	0.79-4.14	0.16	1.77	0.76-4.11	0.19	1.82	0.74-4.49	0.19
*CDH1*	Negative	405 (94.63)	392 (91.59)	1.00			1.00			1.00		
	Positive	23 (5.37)	36 (8.41)	0.62	0.36-1.06	0.08	0.59	0.34-1.02	0.06	0.65	0.36-1.19	0.16
*CDKN2A*	Negative	419 (97.90)	424 (99.07)	1.00			1.00			1.00		
	Positive	9 (2.10)	4 (0.93)	2.28	0.70-7.45	0.17	2.26	0.68-7.51	0.18	1.99	0.56-7.04	0.29
*DAPK1*	Negative	322 (75.23)	385 (89.95)	1.00			1.00			1.00		
	Positive	106 (24.77)	43 (10.05)	**2.95**	**2.01-4.33**	**<0.0001**	**2.93**	**1.98-4.33**	**<0.0001**	**2.95**	**1.94-4.49**	**<0.0001**
*IGF2*	Negative	334 (78.04)	389 (90.89)	1.00			1.00			1.00		
	Positive	94 (21.96)	39 (9.11)	**2.81**	**1.88-4.19**	**<0.0001**	**2.63**	**1.75-3.96**	**<0.0001**	**2.54**	**1.65-3.92**	**<0.0001**
*MGMT*	Negative	365 (85.28)	389 (90.89)	1.00			1.00			1.00		
	Positive	63 (14.72)	39 (9.11)	**1.72**	**1.13-2.63**	**0.01**	1.48	0.96-2.27	0.08	1.82	1.00-2.60	0.05
*MINT31*	Negative	409 (95.56)	423 (98.83)	1.00			1.00			1.00		
	Positive	19 (4.44)	5 (1.17)	**3.93**	**1.45-10.62**	**0.01**	**4.02**	**1.47-10.99**	**0.01**	**4.27**	**1.52-12.05**	**0.01**
*MLH1*	Negative	412 (96.26)	420 (98.13)	1.00			1.00			1.00		
	Positive	16 (3.74)	8 (1.87)	2.04	0.86-4.82	0.11	1.94	0.81-4.65	0.14	1.72	0.68-4.34	0.25
*NEUROG1*	Negative	363 (84.81)	401 (93.69)	1.00			1.00			1.00		
	Positive	65 (15.19)	27 (6.31)	**2.66**	**1.66-4.26**	**<0.0001**	**2.61**	**1.62-4.21**	**<0.0001**	**2.57**	**1.55-4.25**	**<0.0001**
*WIF1*	Negative	354 (82.71)	392 (91.59)	1.00			1.00			1.00		
	Positive	74 (17.29)	36 (8.41)	**2.28**	**1.49-3.48**	**<0.0001**	**2.26**	**1.47-3.48**	**<0.0001**	**2.44**	**1.53-3.87**	**<0.0001**
MRS_10	Low	172 (40.18)	319 (74.53)	1.00			1.00			1.00		
	Medium	177 (41.36)	87 (20.33)	**3.77**	**2.75-5.18**	**<0.0001**	**3.66**	**2.66-5.05**	**<0.0001**	**3.85**	**2.72-5.45**	**<0.0001**
	High	79 (18.46)	22 (5.14)	**6.67**	**4.01-11.06**	**<0.0001**	**6.41**	**3.84-10.71**	**<0.0001**	**6.51**	**3.77-11.27**	**<0.0001**
											*Ptrend*	<0.0001
	Medium or High	256 (59.81)	109 (25.47)	**4.36**	**3.26-5.83**	**<0.0001**	**4.22**	**3.14-5.66**	**<0.0001**	**4.39**	**3.19-6.05**	**<0.0001**

CI: confidence interval; CRC: colorectal cancer; MRS: methylation risk score; OR: odds ratio.

^a^ According to the Youden index, a 1% level of methylation was used as the cut-off value for *APC*, *CDH1* and *IGF2*, and a 0% level of methylation was used as the cut-off value for *CDKN2A*, *DAPK1*, *MGMT*, *MINT31*, *MLH1*, *NEUROG1* and *WIF1*. The positive indicates methylated status and the negative indicates unmethylated status. ^b^ The rates represent the percentages of all cases and controls, respectively. ^c^ ORs adjusted for age, gender and BMI. ^d^ ORs adjusted for age, gender, BMI, occupational physical activity, smoking, and consumption of coarse grains, fish stewed with brown sauce, fried food, leftovers and pork.

*P* values < 0.05 are in bold.

### Subgroup analysis

For MRS_10, the MRS_High and the MRS-Medium groups conferred an increased risk of CRC among both men and women, although the effect was attenuated in women compared with men (Supplementary Table 2). Additionally, significant associations were observed between the methylation of *DAPK1*, *IGF2*, *NEUROG1* and *WIF1* and CRC risk in both men and women. However, the methylation of *MGMT* and *MINT31* only displayed statistically significant associations with CRC in men.

The methylation of *DAPK1*, *IGF2*, *NEUROG1* and *WIF1* was associated with CRC risk in both the younger (<60 years) and older groups (≥60 years) (Supplementary Table 3), whereas the associations between the methylation of *MGMT* and *MINT31* and CRC risk were significant only in the younger participants. Clearly significant associations were observed between the MRS-High and the MRS_Medium groups and CRC risk among both the younger and the older participants.

According to body-mass index (BMI), the MRS_High and the MRS-Medium groups conferred a similarly increased risk of CRC in both normal weight or underweight subjects (<24) and overweight or obese subjects. For individual gene-specific methylation, the methylation of *DAPK1*, *IGF2*, *NEUROG1* and *WIF1* was associated with CRC risk in both of the normal weight or underweight group (<24) and the overweight or obese group (≥24) (Supplementary Table 4), whereas the associations between the methylation of CDH1, MGMT and MINT31 and CRC risk were significant only in the overweight or obesity participants.

### Interactions between EF and DNA methylation

The significant interaction between increased intake of fruit and the methylation of *IGF2* displayed an antagonistic effect on the risk of CRC. Alternatively, the significant interaction between increased consumption of fish stewed with brown sauce and the methylation of *CDH1* displayed a synergistic effect on the risk of CRC. Furthermore, the interactions between increased consumption of coarse grains and the methylation of *IGF2* and between increased consumption of pork and the methylation of *NEUROG1* demonstrated a marginally significant antagonistic effect on the risk of CRC (Table 3).

**Table 3:** Effects of interactions between environmental factors and gene methylation on the risk of colorectal cancer

Gene methylation	Environmental factor				Gene methylation	Environmental factor			
	Consumption of fruits (times/week)					Consumption of coarse grains (g/week)			
	<2	≥2	Interaction			<200	≥200	Interaction	
*IGF2*	OR_eg_ (95% CI)		OR_i_^a^ (95% CI)	*P*	*IGF2*	OR_eg_ (95% CI)		OR_i_^a^ (95% CI)	*P*
Negative	1	0.91(0.74-1.11)			Negative	1	0.69(0.57-0.84)		
Positive	3.72(2.08-6.66)	1.64(1.00-2.71)	**0.35(0.14-0.88)**	**0.0248**	Positive	5.44(2.67-11.08)	1.50(0.95-2.38)	0.44(0.18-1.10)	0.0787
	**Consumption of stewed fish with brown sauce (times/week)**					**Consumption of pork (g/week)**			
	**<1**	**≥1**	**Interaction**			**<250**	**≥250**	**Interaction**	
*CDH1*	OR_eg_ (95% CI)		OR_i_^a^ (95% CI)	*P*	*NEUROG1*	OR_eg_ (95% CI)		OR_i_^a^ (95% CI)	*P*
Negative	1	1.50(1.15-1.95)			Negative	1	1.13(0.91-1.41)		
Positive	0.31(0.15-0.66)	2.00(0.81-4.96)	**3.82(1.11-13.15)**	**0.0333**	Positive	3.33(1.62-6.86)	1.90(1.06-3.39)	0.39(0.14-1.07)	0.0671

CI: confidence interval; OR_eg_: odds ratio for the combined effect of the gene methylation pattern and the environmental factor; OR_i_: odds ratio for the interaction between the gene methylation pattern and the environmental factor.

^a^ The OR_i_ was adjusted for age, gender and BMI.

*P* values < 0.05 are in bold.

### Pyrosequencing verification

For *DAPK1* and *MLH1*, the methylation status obtained via MS-HRM was compared with the mean methylation level based on quantitative pyrosequencing and the results indicated that the MS-HRM results were well confirmed by the pyrosequencing results (Supplementary Figure 1). The Spearman correlation coefficients between these two methylation assessment techniques were high (r=0.7147, 95% CI, 0.6145 to 0.8077, *P*<0.0001 for *DAPK1*; r=0.6089, 95% CI, 0.3972 to 0.7392, *P*<0.0001 for *MLH1*), and the AUC was 0.8699 (95% CI, 0.7919 to 0.9479, *P*<0.0001) for *DAPK1* and 0.9113 (95% CI, 0.8445 to 0.9782, *P*<0.0001) for *MLH1*. Additional results about the Bland-Altman plots are provided in the Supplementary Materials.

### Performance of the MRS_10 model

**Figure 1:**
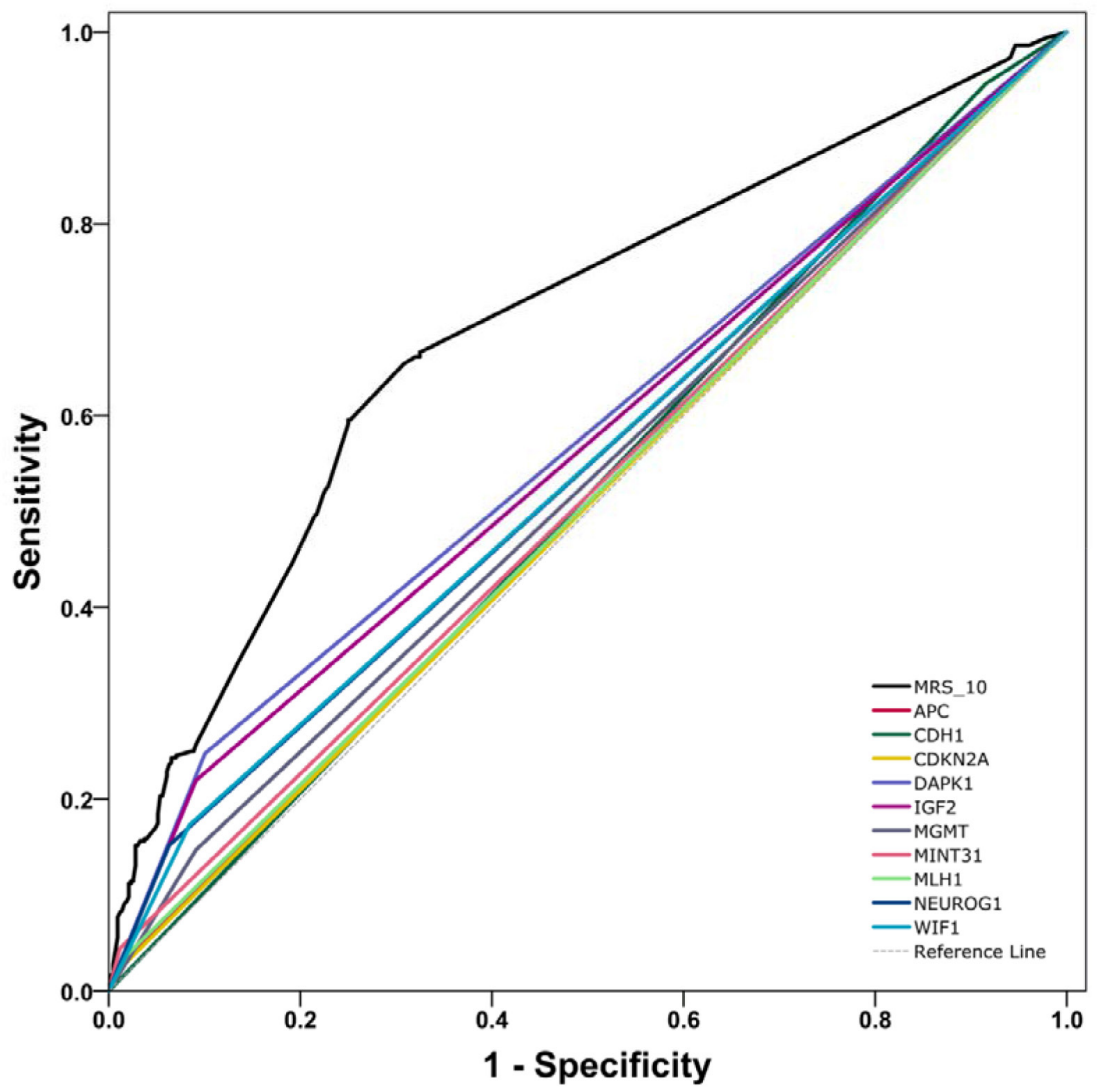

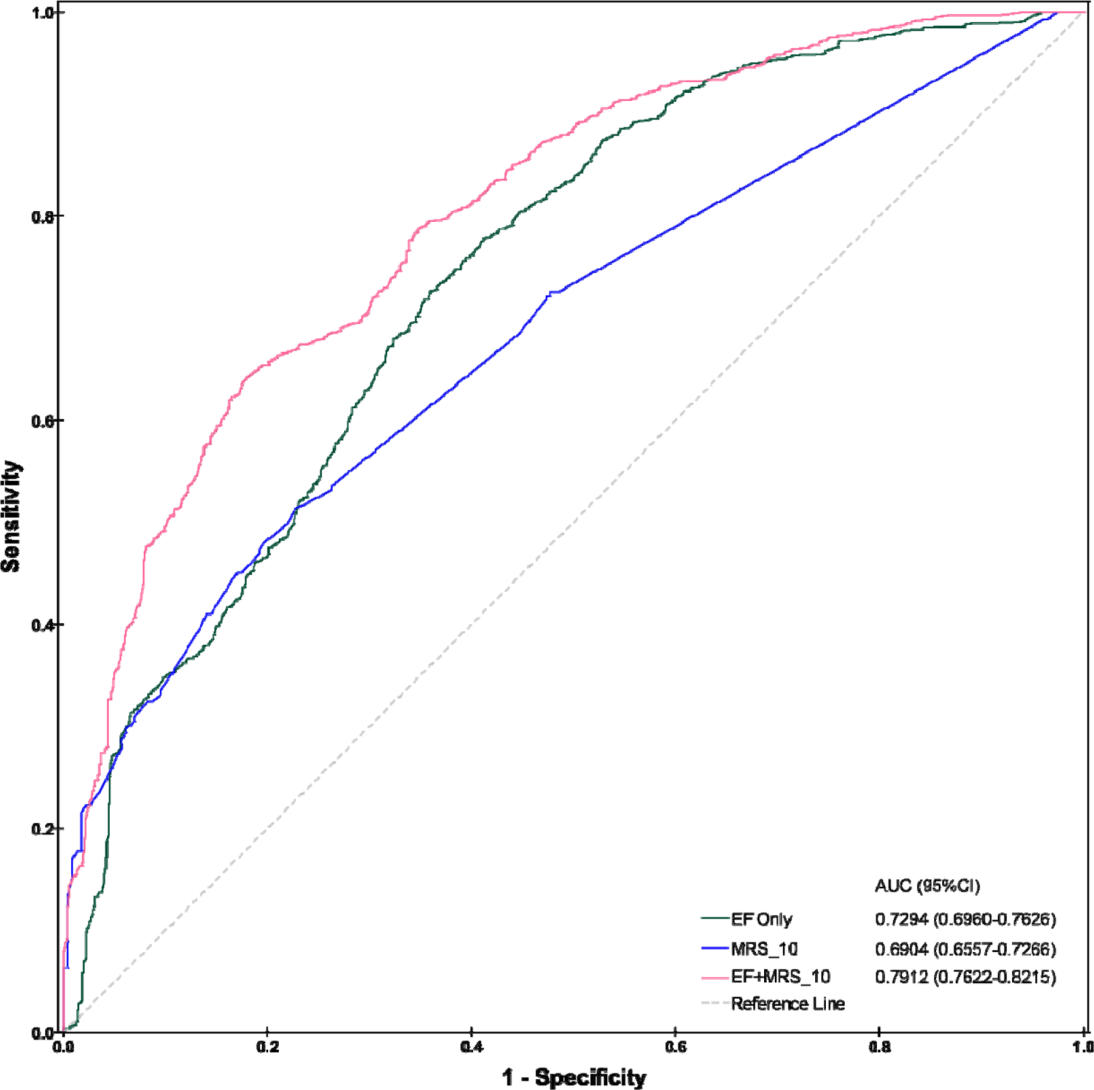

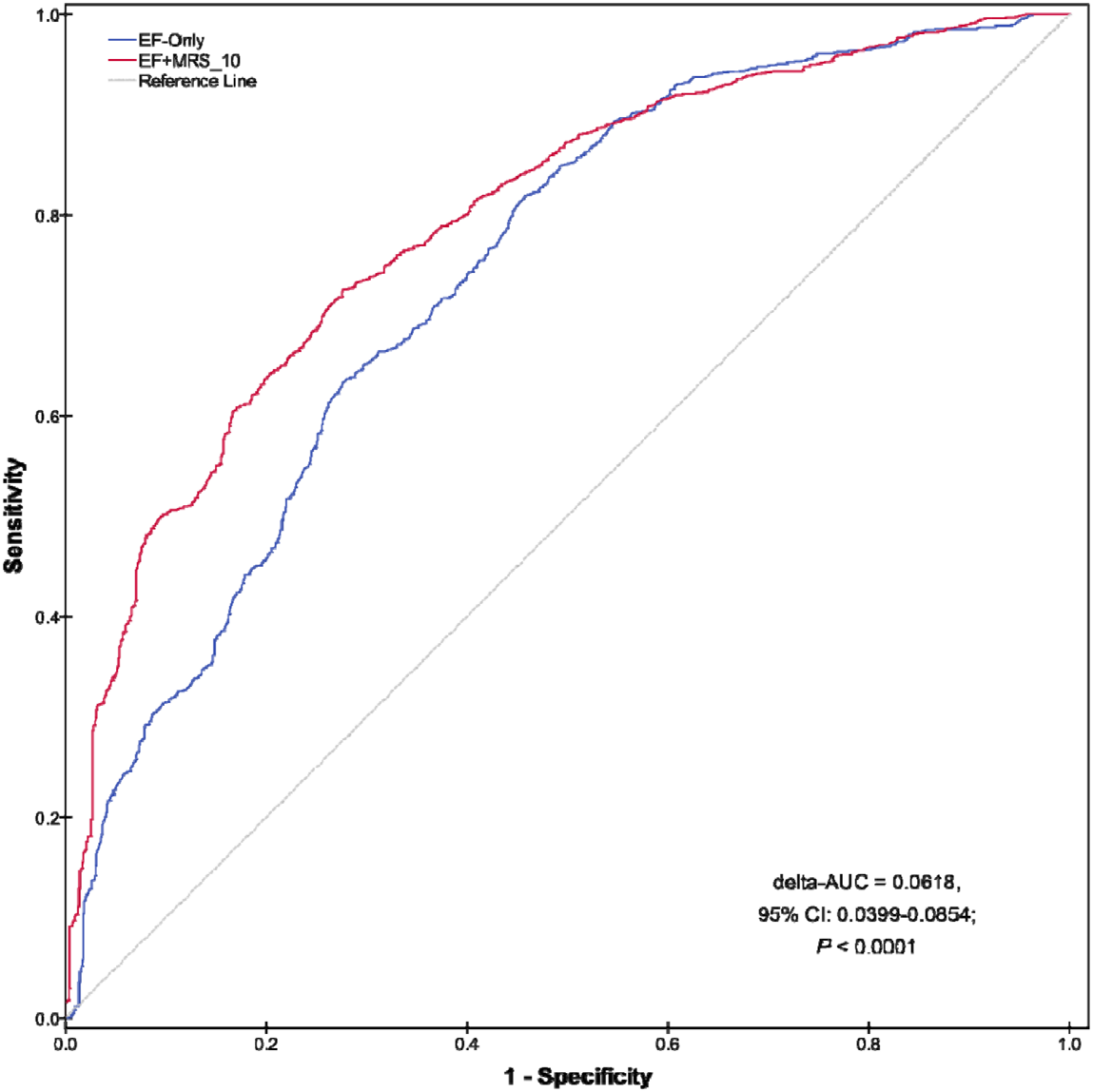
Receiver operating characteristic (ROC) curves and the corresponding area under the curves (AUC) analyses of prediction models of CRC risk **(A)** The MRS_10 model versus any single gene methylation pattern. **(B)** Comparisons of the prediction accuracy by the EF-only model, the MRS_10 model and the combined EF and MRS_10 model. The straight dotted line (reference line), corresponding to an AUC of 0.5, indicates that the model is no more accurate than random classification in predicting CRC risk. **(C) The combined EF and MRS_10 model versus the EF-only model.** ROC curves and AUC analyses were computed for a reference prediction model (blue) and for an extended model (red) including additional risk predictors. Indicated are the increases in AUC (delta-AUC) obtained by adding the additional predictors.

We developed two basic models (including the MRS_10 model and the EF-only model) and a combined model (the combined EF and MRS_10 model). The AUC for the MRS_10 model was 69.04% (95% CI, 65.57 to 72.66%, *P*<0.0001), which represented significantly higher discrimination accuracy than any individual gene methylation (Figure 1). The AUC for the EF-only model was 72.94% (95% CI, 69.60 to 76.26%, *P*<0.0001). Notably, the combined EF and MRS_10 model yielded an AUC of 79.12% (95% CI, 76.22 to 82.15%, *P*<0.0001), representing an increase of 6.18% (95% CI, 3.99 to 8.54%, *P*<0.0001) compared to the EF-only model. Based on the NRI, the IDI and the AUC difference, the improvement of adding MRS_10 to the EF-only model was statistically significant (Supplementary Table 5). The NRI and the IDI for addition of the MRS_10 to the EF-only model was 14.72% (95% CI, 8.92 to 20.52%, *P*<0.0001) and 9.30% (95% CI, 7.36 to 11.23%, *P*<0.0001), respectively.

### Relationships between EF and DNA methylation

Based on comparisons of the higher MRS group (combining the MRS-Medium and MRS-High group) with the MRS-Low group, smoking increased the risk of MRS_10 in all the subjects and cases but not in the controls (Supplementary Table 6). Considering the methylation of individual genes, we found that smoking and high consumption of leftover were significantly associated with *DAPK1* hypermethylation in all the subjects; the associations remained marginally significant in the cases but not in the controls. High consumption of pork was significantly associated with *NEUROG1* hypermethylation in all the subjects and controls but not in the CRC cases. High consumption of fish stewed with brown sauce was significantly associated with *CDH1* hypermethylation only in the CRC cases.

## DISCUSSION

This study found that MRS_10 were strongly associated with the risk of CRC, indicating a positive relationship between leukocyte-derived DNA methylation of the panel of 10 genes and CRC risk. By using of MRS_10, compared with the MRS-Low group, subjects with MRS-High and MRS-Medium showed 6.51-fold and 3.85-fold increased risk of CRC, respectively. Stratified analyses demonstrated that these significant associations retained in both men and women, younger and older, and normal weight or underweight and overweight or obese subjects.

The findings demonstrated that the DNA methylation pattern in peripheral blood leukocytes is a detectable biomarker for CRC risk assessment. This observation is similar to those results for other cancers reported in previous studies [31–34] suggesting that DNA methylation alterations in peripheral blood are potential biomarkers for risk prediction.

The gene involved in the study illustrated a wide range of cancer related cellular events, for example, *CDH1* encoded a member of the family of cell adhesion, and abrogation in its expression have been involved in unregulated growth and invasion of adjacent tissues in carcinogenesis [35]. Likewise, as a tumor suppressor gene, *CDKN2A* played an important control role in cell cycle regulating during the G1 phase [36]. *DAPK1* was a positive mediator of apoptosis which executed juncture of cell death signaling, and its loss or inactivation has been linked to human tumor [37]. *WIF1* was a secreted inhibitory factor of Wnt pathway, which played a pivotal role in blockade of Wnt signaling and induced apoptosis in colorectal cancer cells [10]. A growing body of evidence suggested that aberrant hypermethylation of the promoter can lead to reduced expression of these genes including *APC* [9], *CDH1* [38], *CDKN2A* [39], *DAPK1* [40], *IGF2* [41], *MGMT* [42], *MLH1* [43], *NEUROG1* [44] and *WIF1* [45]. In contrast to the methylation status of a single gene, the MRS, which summarized the data for the methylation of multiple genes, might demonstrate systematically altered gene methylation profiles in subjects and might more comprehensively represent the susceptibility of an individual to CRC. In addition, sensitivity analyses by omitting each individual locus showed a robust association between the MRS and the risk of CRC (Supplementary Table 7).

The exact mechanism underlying the alterations in the methylation of peripheral blood-derived DNA among individuals who are susceptible to CRC remains unclear. Alterations in leukocyte-derived DNA methylation may reflect a response of the hematopoietic system to tumorigenesis and may be partially explained by systemic differences in the methylation signatures of leukocyte subpopulations during tumorigenesis [46]. The leukocyte-derived DNA methylation profiles represented the overall methylation status; nevertheless, isolation of specific cell subpopulations is difficult in epidemiologic studies [47]. Additionally, in the present case-control study, it is not possible to determine the etiologically relevant time windows of DNA methylation or confirm the temporal sequence of DNA methylation and CRC occurrence. Although we cannot definitely determine whether these methylation alterations in peripheral blood-derived DNA represent an early response of the hematologic system to the presence of tumor cells or appear before tumor development [31], the observed significant association between gene-specific DNA methylation in leukocytes and CRC risk is likely to be useful in identifying the population at high risk of CRC.

Importantly, circulating tumor cell DNA is unlikely to interfere with the results of leukocyte-derived DNA methylation because the number of circulating tumor cells is negligible and the effects of their aberrant methylation can be excluded [32, 48]. Another potential source of support of our findings is that accumulating studies have shown no significant correlation between tissue and blood DNA methylation [49, 50]. We also compared the DNA methylation levels of six genes in peripheral-blood leukocytes and colorectal tumor tissues from 217 CRC patients; no statistically significant correlations were observed (Supplementary Table 8).

The potential biological mechanism for EF interfering with DNA methylation processes in carcinogenesis is complex and variable. Several researchers have suggested the plausible mechanisms, including that DNA methylation requires methyl group donors such as S-adenosylmethionine (SAM), while environmental factors may affect SAM synthesis and alter DNA methyltransferase (DNMT) activity [51]. In this study, we found that smoking was associated with DNA hypermethylation, which was consistent with several previous studies [52, 53]. Smoking can induce DNMT1 overexpression and subsequently result in hypermethylation of the promoter of tumor suppressor genes, which could lead to tumorigenesis [54]. The molecular mechanism underlying the link between consumption of pork or stewed fish with brown sauce and DNA methylation remains unclear. Saturated fatty acids and heterocyclic amines, which form during the high-temperature cooking of pork and fish, may partly account for the aberrant hypermethylation [55, 56]. Alteration of DNA methylation is a gradual and reversible process, thus, there is a critical window of opportunity through which we might inhibit or reverse the process and counteract cancer by making changes in diet and lifestyle [57].

We initially used MS-HRM to assess DNA methylation status. MS-HRM technology has been shown to reliably and accurately evaluate low-level methylation [58]. By comparing the estimates of methylation of *DAPK1* and *MLH1* based on MS-HRM and pyrosequencing, we found that the MS-HRM results were highly consistent with the pyrosequencing results. This consistency was also supported by a previous study that analyzed *APC* and *CDKN2A* methylation and found a high correlation between the results from these two techniques [59]. Only a small subset of genes and participants were analyzed via pyrosequencing because of its high cost. From a cost perspective, the assessment of peripheral blood-derived DNA methylation via MS-HRM might be an efficient strategy for the early detection of individuals who are at high risk for CRC.

In this study, we developed the weighted MRS_10 that combined these 10 genes in a leukocyte-derived DNA methylation marker panel and found that the AUCs for the MRS_10 model and the combined EF and MRS_10 model reached 0.6904 and 0.7912, respectively. Compared to the EF-only model with a similar AUC to those reported in previous studies (AUCs ranging from 0.61 to 0.76) [60–69], adding the MRS_10 significantly improved the discriminatory performance, as demonstrated by a 0.0618 units of AUC gain. However, the combined EF and MRS model is needed to be further validated in future prospective studies. To evaluate whether adding the MRS_10 to the EF-only model improved risk prediction performance, we assessed the accuracy of the combined model in classifying individuals as cases or controls based on the NRI and the IDI, which are useful statistics that have gained increasing acceptance for the evaluation of new biomarkers and risk models [70, 71]. Adding MRS_10 to the EF-only model resulted in the reclassification of 14.72% of the subjects into more accurate risk categories. This advancement might improve the selection of those who require more frequent screening and shorter follow-up intervals. However, NRI is sensitive to arbitrary cut-off values [72]. Therefore, we reset the cut-off points to 0.3, 0.4, 0.6 or 0.7 and found similar NRIs (Supplementary Table 9), which implied that the discriminatory improvement was robust across the cut-off values used in our study.

Our study has several additional limitations. First, the current panel of gene methylation sites incorporated into the calculation of MRS_10 might not be ideal because we have identified the genes from published studies rather than next-generation sequencing, which would likely reveal additional methylation biomarkers. Therefore, the present MRS_10 models in this study must be updated. Another potential limitation of this study is the restriction of EFs included in the combined EF and MRS_10 model. The age and gender matching design inherent to our present study removes two CRC-associated variables. Additionally, the discriminatory performance of the combined model might be improved by including additional factors such as colonoscopy or sigmoidoscopy findings.

In summary, the MRS_10 combining a panel of 10 gene-specific DNA methylation in leukocytes seemed to be a promising and robust risk prediction tool and might be a useful marker of susceptibility for CRC. The MRS_10 may be useful for the identification of individuals who are at high risk of developing CRC. However, our results should be further validated in future studies.

## MATERIALS AND METHODS

### Study population

We included primary sporadic CRC cases diagnosed at the Third Affiliated Hospital (from June 2004 to May 2005 and May 2007 to January 2008) and the Second Affiliated Hospital of Harbin Medical University (from October 2010 to December 2011) in Harbin, China. Cancer-free controls were selected contemporaneously from the Second Affiliated Hospital of Harbin Medical University by individual matching each case according to gender and age (±2 years). All participants were Chinese. All CRC cases were newly diagnosed, histologically confirmed, and alive at the time of initial contact; and the exclusion criteria included subjects with metastatic colorectal carcinoma, adenomatous polyposis coli, or a family history of CRC in first-degree relatives according to the Amsterdam criteria [73]. The participants were interviewed face-to-face to complete a structured standard questionnaire, which was partially adopted from the report by Shu et al [74]. The questionnaire queried information on demographic characteristics and potential risk factors for CRC, including family history, smoking, alcohol drinking, occupational physical activity, and diet consumption. Dietary consumption over the past year was assessed using a validated food frequency questionnaire (FFQ) [74]. The FFQ included 9 major food groups, which represent most of the common foods in Northeast China. The food items included coarse grains, dairy products, fish stewed with brown sauce, fried food, fresh fruits, green vegetables, leftover, pork, and soybean products.

Through professional training, the investigators made efforts to reduce recall and investigation bias. All study subjects gave informed consent, and appropriate ethical approval for sample collection was obtained from the Ethics Committee of Harbin Medical University prior to the study. Peripheral blood (5 milliliters) was donated before chemotherapy or adjuvant radiotherapy and was stored in a freezer. The sample size estimation was presented in the Supplementary Materials. In total, 553 eligible patients were recruited; 32 did not complete the questionnaire, and another 19 did not provide sufficient blood samples. Therefore, 502 extracted DNA samples were available for the CRC cases, with a response rate of 90.78%. Similarly, 1,210 subjects were eligible as controls, and 1,083 provided complete questionnaire data and blood samples with a response rate of 89.50%. Because eligible matched controls could not be found for 45 cases, 457 controls were ultimately enrolled.

### Genomic DNA extraction and bisulfite modification

DNA was extracted from buffy coats using the QIAamp DNA Blood Mini Kit (Qiagen, Hilden, Germany) and was then bisulfite-modified using the EpiTect Plus DNA Bisulfite Kit (Qiagen) according to the manufacturer’s protocols. Detailed methods are provided in the Supplementary Materials.

### Methylation-sensitive high-resolution melting (MS-HRM) assays

Fourteen CRC-related genes, including tumor suppressor genes such as *APC*, *CDH1*, *CDKN2A* (also known as *p16*), *CDKN2B* (also known as *p15*), *DAPK1*, *GSTP1*, *MGMT*, *MLH1*, *PTEN* and *WIF1* and CpG island methylator phenotype-related markers such as *IGF2*, *APBA1* (also known as *MINT1*), *MINT31* and *NEUROG1*, were selected according to review of literatures. Eight primer pairs were selected from previously published studies [75–81]; the remaining primers were designed using Methprimer software [82]. The primer sequences used are listed in Supplementary Table 10. The PCR mixture consisted of a total volume of 10 μl containing 2× LightCycler 480 High Resolution Melting Master Mix (Roche Applied Science, Mannheim, Germany), 3 mM MgCl2, 0.2-0.4 μM of each primer and approximately 10 ng of bisulfite-modified template DNA. PCR amplification and MS-HRM analyses were performed using the LightCycler 480 platform (Roche), and the resulting data were analyzed using software module of Gene Scanning (Roche). A set of methylation standards (100, 25, 10, 5, 2, 1, and 0% methylated DNA) were prepared by mixing commercially available methylated and unmethylated DNA (Zymo Research); these standards were used to semi-quantitatively measure the DNA methylation level in the samples. Normalized melting curves of MS-HRM assays for each gene were shown in Supplementary Figure 2. In addition, a blank control (non-template control) sample was included in each batch, and all reactions were performed in duplicate. A third trial was conducted for the samples that presented inconsistent results between the two trials.

Two investigators (Y.L. and Y.W.) blinded to outcome and other predictive variables assessed the MS-HRM results, and discrepancies were resolved by discussion and consensus with another investigator (Y.Z.). The DNA methylation pattern was first tested in 167 cases and their matched controls. Four genes displaying no abnormal methylation (*PTEN*, *GSTP1*, *APBA1* and *CDKN2B*) were tested no further and were excluded from the analysis. Finally, the other 10 genes were assessed in all 457 cases and their matched controls.

### Pyrosequencing verification

To verify the results of MS-HRM, we performed pyrosequencing in a subset of the samples (further details are provided in the Supplementary Materials and the primer sets are listed in Supplementary Table 11).

### Methylation risk score (MRS) computation

First, we coded each gene methylation status as 0 or 1 for non-methylation or methylation, respectively, according to the optimal cut-off value as determined by the Youden index (J = max {sensitivity + specificity – 1}) using the receiver operating characteristic (ROC) curve analysis. Then, we computed the weighted MRS for each individual by multiplying the methylation status by the *β*-coefficients for each gene included in the model and then dividing by the number of genes included in the model. Simply, the weighted MRS was computed using the following equation:

$$\mathrm{MRS}=\frac{\beta_{1}x_{1}+\beta_{2}x_{2}+\ldots +\beta_{i}x_{i}+\beta_{k}x_{k}}{k}$$

Where *βi* is the *β*-coefficient for gene *i*, *xi* is the methylation status of the same gene *i* (0 or 1), and *k* is the total number of genes included in the model. According to the formulation listed above, we generated MRS_10 comprehensively considering the methylation status of all the 10 genes. Using the MRS_10 model, the subjects were classified as follows: MRS-Low, predicted probability ≤0.5; MRS-Medium, 0.5< predicted probability ≤0.7; or MRS-High, predicted probability >0.7.

### Missing data analysis and imputation

Definitions of questionnaire-derived variables are provided in the Supplementary Materials. All questionnaire-derived variables were analyzed via missing value analysis and were imputed via multiple imputation (further details are provided in the Supplementary Materials).

### Model development and performance

We selected CRC-associated risk factors by using of multivariable logistic regression model with backward conditional selection method (*P* values of 0.05 and 0.10 were specified as the thresholds for entry and removal of variables, respectively), and we further included gender and age in the final model. Interactions between environmental factors (EF) and gene methylation were also explored, but we did not included interaction terms in the models because interaction terms rarely add to the predictive ability of the model [83, 84]. A multivariable logistic regression model was used to fit the CRC risk prediction models: Model 1 was the MRS_10 model; Model 2 was an EF-only model, which exclusively contained questionnaire-derived risk factors; and Model 3 was a combined model incorporating EF and MRS_10. For each logistic regression model, the regression coefficient estimates were corrected according to the pooled imputed datasets. The estimates of regression coefficients for each model were listed in Supplementary Table 12.

To assess the discriminatory accuracy of each model, we generated ROC curves and calculated the areas under ROC curves (AUCs). The 95% CI for the AUC were estimated using the bootstrapping method (1000 replicates). We used the method of DeLong [85] to assess the differences in the AUC between the models. We further calculated the categorical net reclassification improvement (NRI), the integrated discrimination improvement (IDI) to evaluate the improvement in the discriminatory accuracy of the model (considering 0.5 as the cut-off point).

### Statistical analysis

The differences in characteristics between the cases and the controls were tested using Student’s *t*-test for continuous variables or the χ2 test for categorical variables. We used both univariable and multivariable logistic regression models to estimate odds ratios (ORs) and 95% confidence interval (CI) for the associations between gene-specific DNA methylation or EF and CRC risk. The effects of interactions between EF and the methylation status at individual genes or MRS_10 on the risk of CRC were evaluated on a multiplicative scale with a product-term coefficient using multivariable logistic regression models. Additionally, to assess the associations between EF and DNA methylation among all the subjects, we used multivariable logistic regression models that included age, gender, BMI and case-control status that allowed us to adjust for the potential influence from case-control status. We also assessed the associations by case and control separately. All statistical tests were two-sided except for the test of the increase in AUC (one-sided) and statistical significance was defined as *P* values of less than 0.05. Missing value analysis, multiple imputations and multivariable logistic regression analysis were performed using SPSS Statistics version 19.0 (IBM, Inc., USA). The 95% CIs for the AUC and the NRI and the IDI were estimated using the pROC package and the PredictABEL package in R software version 2.15.3, respectively. All other statistical analyses were performed using SAS software version 9.1 (SAS Institute Inc., USA).

## SUPPLEMENTARY MATERIALS FIGURES AND TABLES




