# Supplementary material for: Multiple gene-specific DNA methylation in blood leukocytes and colorectal cancer risk: a case-control study in China
Source: Oncotarget. 2017 May 22;8(37):61239–52. doi: 10.18632/oncotarget.18054 (PMC5617420; doi:10.18632/oncotarget.18054)
Supplement: Supplementary file 1 [file oncotarget-08-61239-s001.pdf]

Multiple gene-specific DNA methylation in blood leukocytes and colorectal cancer risk: a case-control study in China

SUPPLEMENTARY METHODS

Sample size

We estimated the sample size according to a logistic regression test assessing whether aberrant DNA methylation in blood leukocytes was associated with the risk of colorectal cancer (CRC). A sample size of 652 participants was needed to achieve 90% power (at the 5% level of statistical significance) to detect odds ratios (ORs) of 1.8 or more with a 20% prevalence in the control group. In addition, taking into consideration incomplete questionnaires and the failure rate for MS-HRM detection, we included about 20% more samples and finally targeted a total sample size of 800 participants.

DNA extraction

After the peripheral blood samples were centrifuged at 1,600 g for 10 minutes to separate plasma, leukocyte-derived DNA was extracted from buffy coats using the QIAamp DNA Blood Mini Kit (Qiagen, Hilden, Germany) according to the manufacturer's protocol. Briefly, buffy coats were resuspended in 400 µl of phosphate-buffered saline. After the addition of 40 µl of proteinase K and 400 µl of buffer AL (Qiagen), the mixture was incubated at 56°C for 10 min, treated with 400 µl of ethanol (99%), and mixed. The mixture was transferred to a QIAamp spin column and centrifuged at 6,000 g at room temperature for 1 minute, followed by washing with buffers AW1 and AW2 and centrifugation. DNA was eluted in 150 µl of buffer AE and was quantified using a NanoDrop 2000c bioanalyzer (Thermo-Fisher, USA). Then, the DNA was divided into aliquots and stored at -80°C.

Bisulfite modification

From each subject, 500 ng of DNA was bisulfite-modified using the EpiTect Plus DNA Bisulfite Kit (Qiagen) according to the manufacturer's protocol. The bisulfite-treated DNA was eluted in 30 µl of elution buffer and quantified using a NanoDrop 2000c bioanalyzer set to RNA as the sample type [1]. Then, the bisulfite-modified DNA sample was diluted to a final concentration of 10 ng/µl and was distributed into three aliquots for storage (-20°C).

Pyrosequencing verification

To verify the results of MS-HRM, we performed pyrosequencing in a subset of the samples. For *DAPK1*, pyrosequencing was performed for quantitative methylation analyses of 48 HRM-positive samples (36 CRC case and 12 control samples that were selected from 106 cases and 43 controls, respectively) and 48 HRM-negative samples (24 CRC case and 24 control samples that were selected from 322 cases and 385 controls, respectively); for *MLH1*, 24 HRM-positive samples (16 CRC case and 8 control samples) and 48 HRM-negative samples (24 CRC case and 24 control samples that were selected from 412 cases and 420 controls, respectively).

The regions analyzed via MS-HRM included 7 and 21 CpG dinucleotide sites for *DAPK1* and *MLH1*, respectively. Subsequently, we designed pyrosequencing primer sets to analyze the corresponding regions for *DAPK1* and *MLH1* using PyroMark Assay Design Software (version 2.0.1.15, Qiagen). PyroPCR was performed using a PyroMark PCR Kit (Qiagen) according to the manufacturer's protocol. The primer sets used are listed in Supplementary Table 11 (the reverse primer in each set was biotinylated). The standard conditions for PyroPCR were as follows: 12.5 µl of PyroMark PCR Master Mix, 2.5 µl of CoralLoad Concentrate, 0.5 µl of each primer (10 pmol/µl) and 2 µl (nearly 20 ng) of bisulfite-modified DNA in a final volume of 25 µl. The PyroPCR thermocycling protocol was as follows: initial PCR activation (95°C for 15 min), 45 3-step cycles (94°C for 30 s, 56°C for 30 s, and 72°C for 30 s) and final extension (72°C for 10 min). For each sample, 10 µl of PyroPCR product was used for pyrosequencing reactions and methylation quantification, which were performed using a PyroMark Q24 Advanced System version 2.0.6 (Qiagen) according to the manufacturer's protocol. The pyrosequencing results were analysed using PyroMark software version 3.0.0 (Qiagen) to obtain a percentage of methylation at each CpG site. In the MS-HRM analysis, we could detect only the mean methylation percentage of the analyzed sequences and could not precisely quantify the methylation percentage of individual CpG sites. Therefore, the mean methylation percentage of all CpG sites based on pyrosequencing was calculated for each analyzed gene, and this value was used for comparison with the results of MS-HRM. The concordance between the methylation levels derived from the two DNA

methylation assessment methods was assessed using Spearman correlation coefficients, ROC curves and AUC analyses. Additionally, we used the Bland-Altman plot as a graphical method to compare the two methods.

Definitions of questionnaire-derived variables

Questionnaire-derived variables considered included: age (<60, ≥60), gender, BMI (<24, ≥24), family history of any cancer other than CRC in first-degree relatives (yes, no), occupational physical activity (blue-collar, white-collar), smoking status (no, yes), and consumption of coarse grains (<50, ~100, ~200, ≥200 g/week), dairy (<1, ≥1 times/week), fish stewed with brown sauce (<1, ≥1 times/week), fried food (<1, ≥1 times/month), fruits (<2, ≥2 times/week), green vegetables (<100, ≥100 g/day), leftovers (<1, ≥1 times/week), pork (<250, ≥250 g/week) and soybeans (<1, ≥1 times/week).

Missing data analysis and imputation

All questionnaire-derived variables were analyzed via missing value analysis (Little's missing completely at random (MCAR) test) to assess whether the pattern of missing data was related to the observed data (MCAR or missing at random (MAR)). The MAR assumption fit our data. Therefore, missing data were imputed using multiple imputation (five imputations) via the expectation-maximization method, with all questionnaire-derived variables (including outcomes) included in the imputation model. We used the method of multiple imputation to impute the missing dataset rather than single imputation because the former is generally considered to be superior to the latter for solving the problem of missing data [2, 3].

Statistical analysis

The sample size was calculated using PASS version 11.0.7 (NCSS LLC., USA). The Bland-Altman plot analysis was performed using MedCalc software version 15.4 (Ostend, Belgium).

SUPPLEMENTARY RESULTS

Missing data and imputation

The characteristics of demographic variables and questionnaire-derived variables of the participants before and after multiple imputations are listed in Supplementary Table 1. Across the 856 subjects, missing data occurred in less than 5% for each questionnaire-derived variable, except for consumption of fruits, green vegetables and dairy. The environmental factors that are missing across the entire dataset included family history (values missing for 24 participants, 2.80% of the samples), occupational physical activity (13, 1.52%), dietary consumption of coarse grains (2, 0.23%), dairy products (206, 24.07%), fish stewed with brown sauce (11, 1.28%), fried food (5, 0.58%), fresh fruits (50, 5.84%), green vegetables (52, 6.07%), leftover (7, 0.82%), pork (24, 2.80%), and soybean products (1, 0.12%).

Pyrosequencing verification

For *DAPK1* and *MLH1*, the methylation status obtained via MS-HRM was compared with the mean methylation level based on quantitative pyrosequencing. Using Bland-Altman plots, we found that for *MLH1*, all examinations of methylation fell within the limits of agreement except for one sample displaying a high methylation level, in which MS-HRM reported a lower methylation percentage than pyrosequencing (Supplementary Figure 1). For *DAPK1*, all samples fell within the limits of agreement except for three samples with relatively high methylation, in which MS-HRM reported lower levels of methylation than pyrosequencing. In general, only among samples with a higher methylation percentage, MS-HRM tended to indicate lower levels of methylation than pyrosequencing. These resluts indicated that the MS-HRM results were well confirmed by the pyrosequencing results.

REFERENCES FOR SUPPLEMENTARY METHODS

1. Laurin N, Celestin F, Clark M, Wilkinson D, Yamashita B, Fregeau C. New incompatibilities uncovered using the Promega DNA IQ chemistry. Forensic science international. 2015; 257:134-141.
2. Lorenzo-Seva U, Ferrando PJ. POLYMAT-C: a comprehensive SPSS program for computing the polychoric correlation matrix. Behav Res Methods. 2015; 47:884-889.
3. Moons KG, Altman DG, Reitsma JB, Ioannidis JP, Macaskill P, Steyerberg EW, Vickers AJ, Ransohoff DF, Collins GS. Transparent Reporting of a multivariable prediction model for Individual Prognosis or Diagnosis (TRIPOD): explanation and elaboration. Annals of internal medicine. 2015; 162:W1-73.

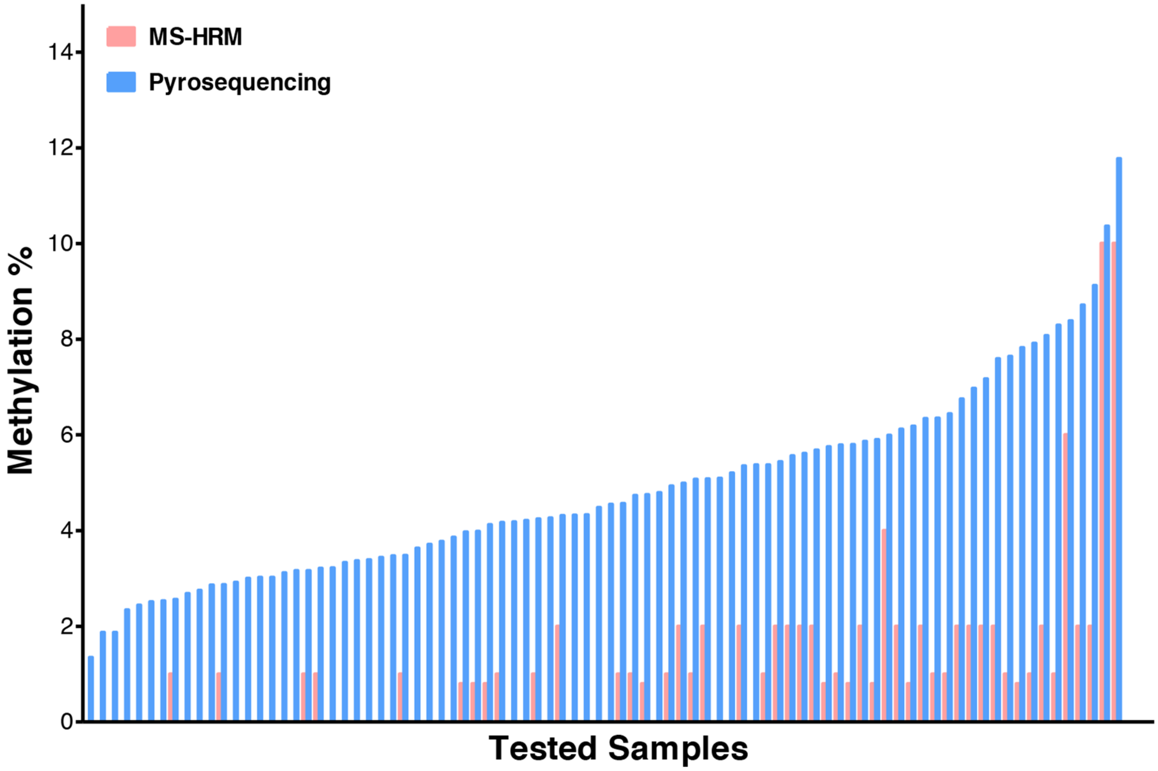
MS-HRM
Pyrosequencing
Methylation %
0
2
4
6
8
10
12
14
Tested Samples

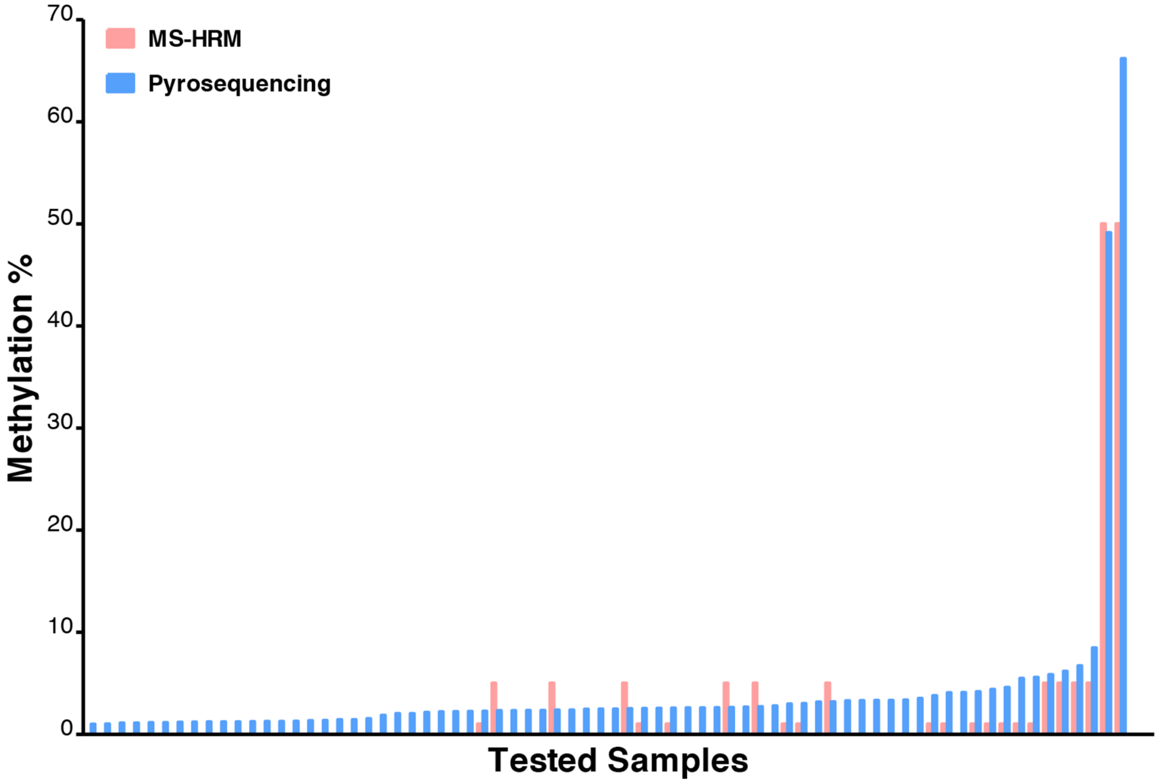
MS-HRM
Pyrosequencing
Methylation %
0
10
20
30
40
50
60
70
Tested Samples

(*Continued*)

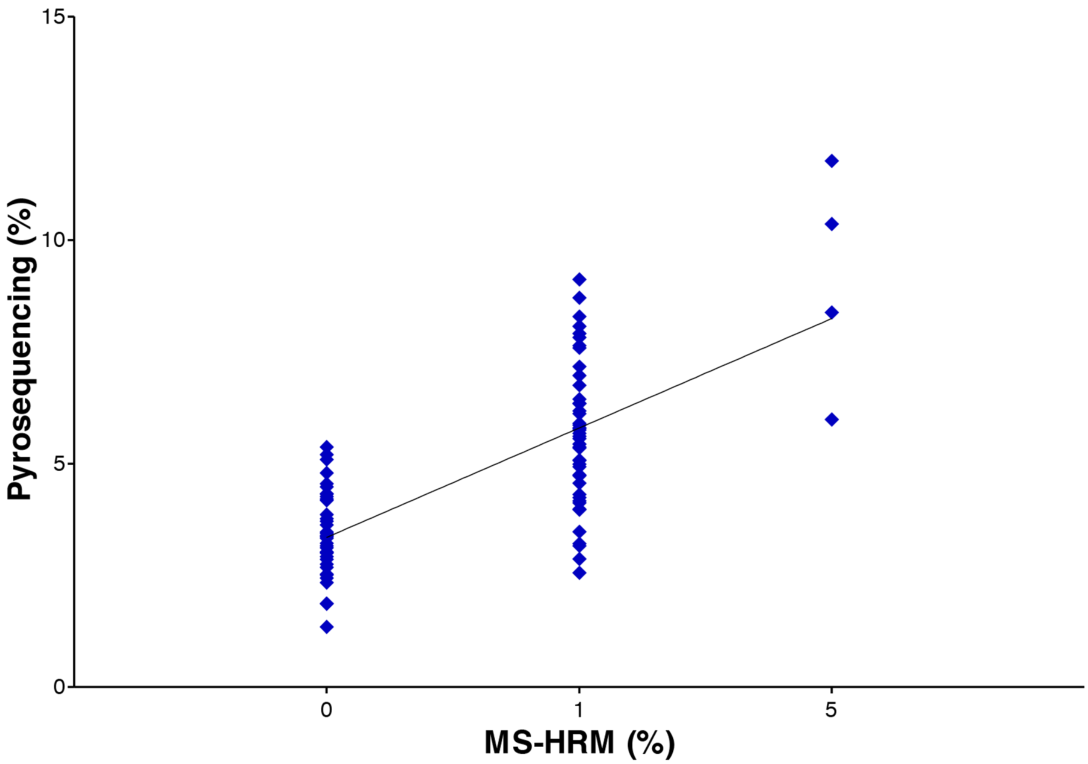
15
10
5
0
Pyrosequencing (%)
0
1
5
MS-HRM (%)

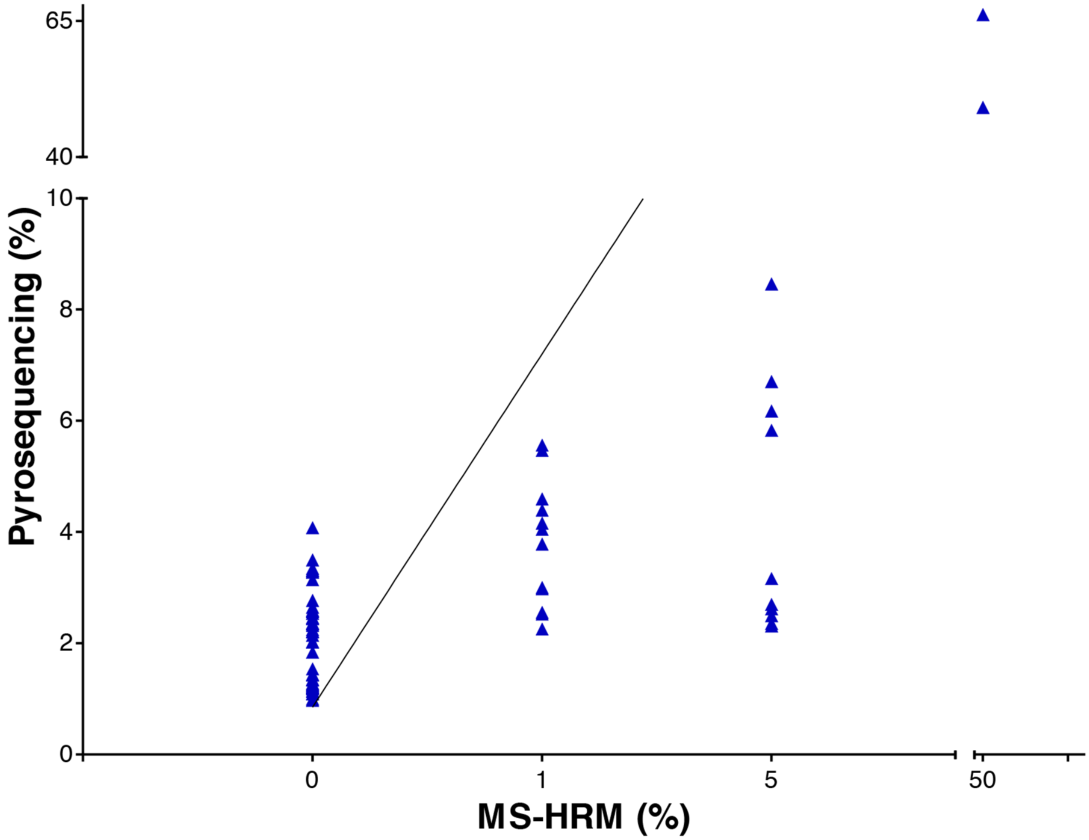
65
40
10
8
6
4
2
0
Pyrosequencing (%)
0
1
5
50
MS-HRM (%)

(*Continued*)

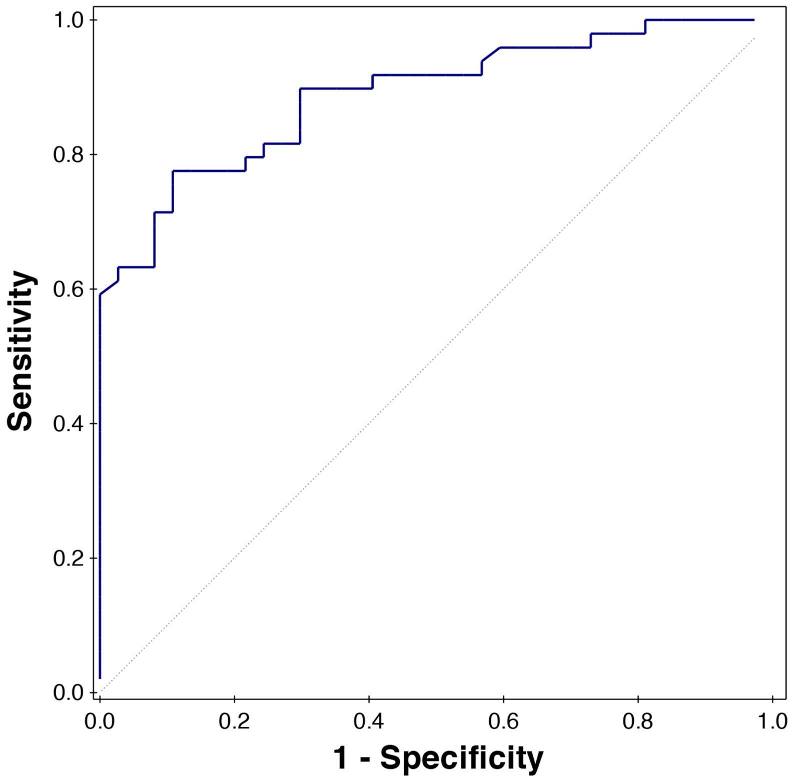
Sensitivity
1 - Specificity
0.0
0.2
0.4
0.6
0.8
1.0

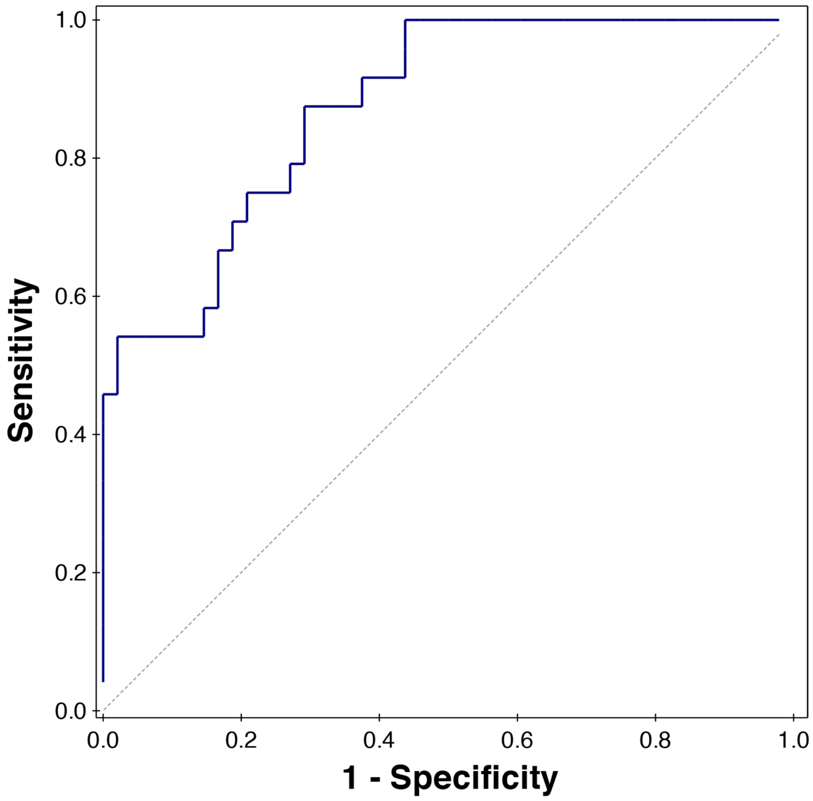
Sensitivity
1 - Specificity
0.0
0.2
0.4
0.6
0.8
1.0

(*Continued*)

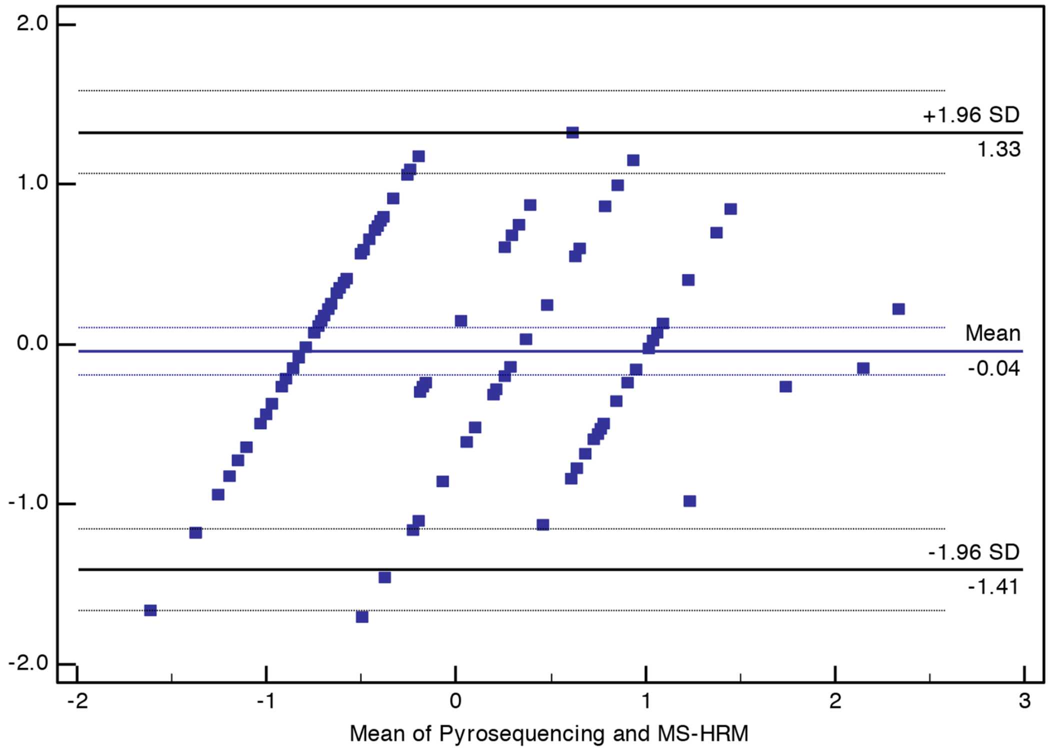

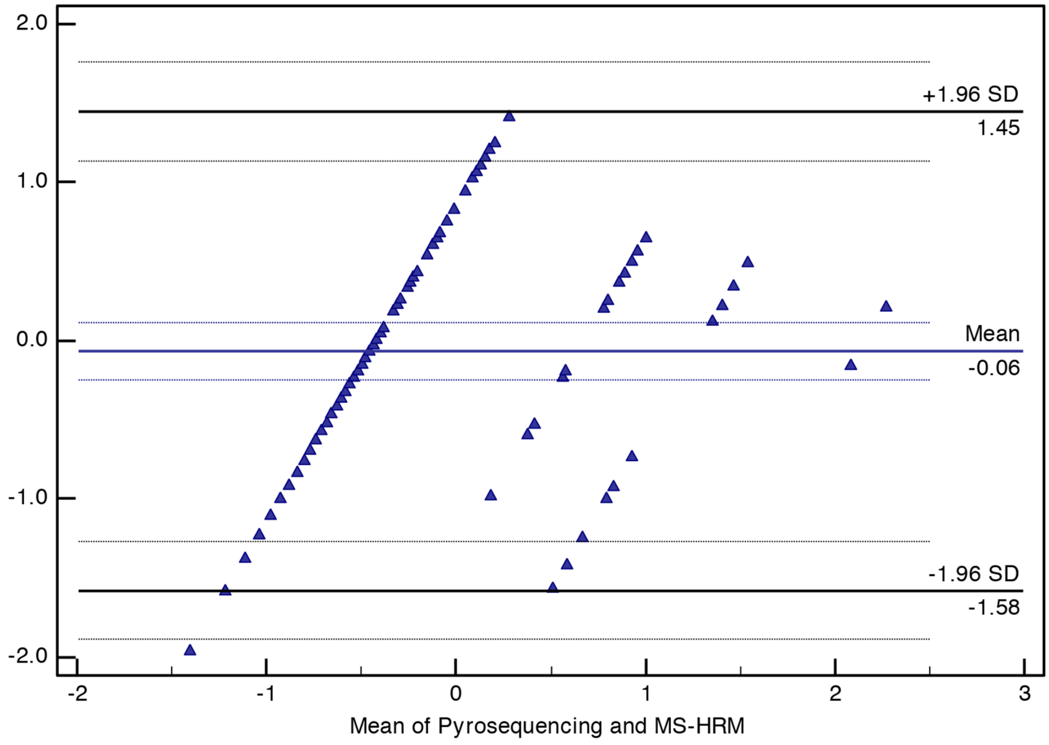

Supplementary Figure 1: Comparisons of the methylation levels of *DAPK1* and *MLH1* obtained by methylation-sensitive high-resolution melting (MS-HRM) and pyrosequencing. Profiles of *DAPK1* **(A)** and *MLH1* **(B)** methylation for each sample. Spearman correlation analyses for *DAPK1***(C)** and *MLH1* **(D)**. Receiver operating characteristic curves (ROC) and corresponding area under the curve (AUC) analyses for *DAPK1* **(E)** and *MLH1* **(F)**. Bland-Altman plots for *DAPK1* **(G)** and *MLH1* **(H).**

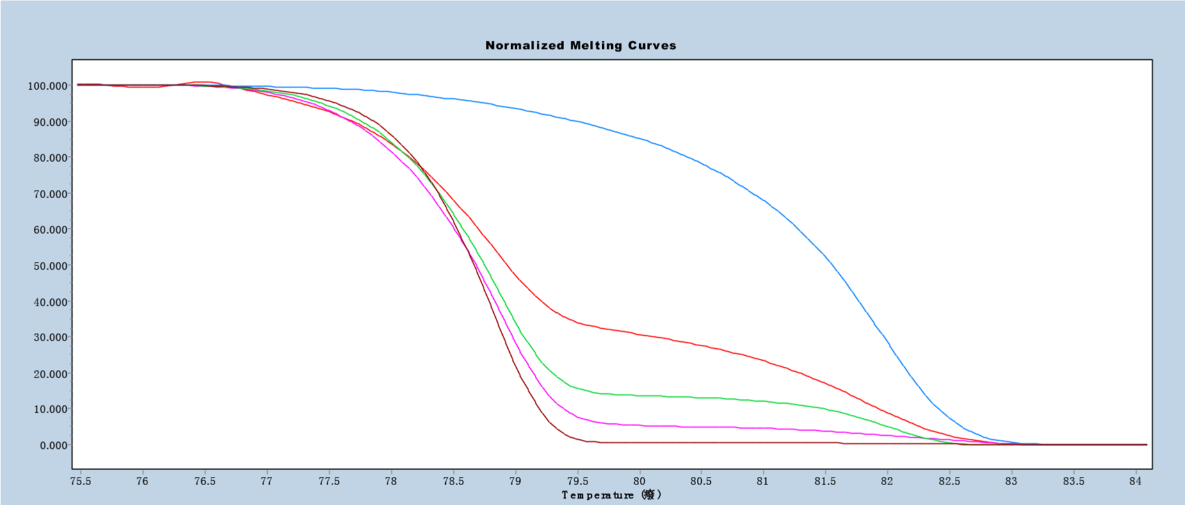
Normalized Melting Curves
100.000
90.000
80.000
70.000
60.000
50.000
40.000
30.000
20.000
10.000
0.000
75.5 76 76.5 77 77.5 78 78.5 79 79.5 80 80.5 81 81.5 82 82.5 83 83.5 84
Temperature

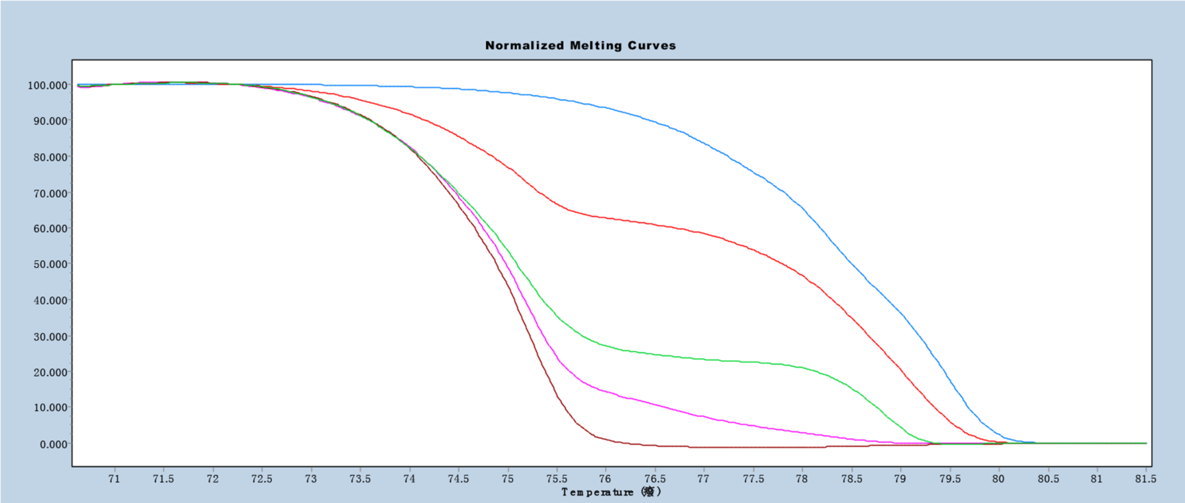
Normalized Melting Curves
100.000
90.000
80.000
70.000
60.000
50.000
40.000
30.000
20.000
10.000
0.000
71 71.5 72 72.5 73 73.5 74 74.5 75 75.5 76 76.5 77 77.5 78 78.5 79 79.5 80 80.5 81 81.5
Temperature

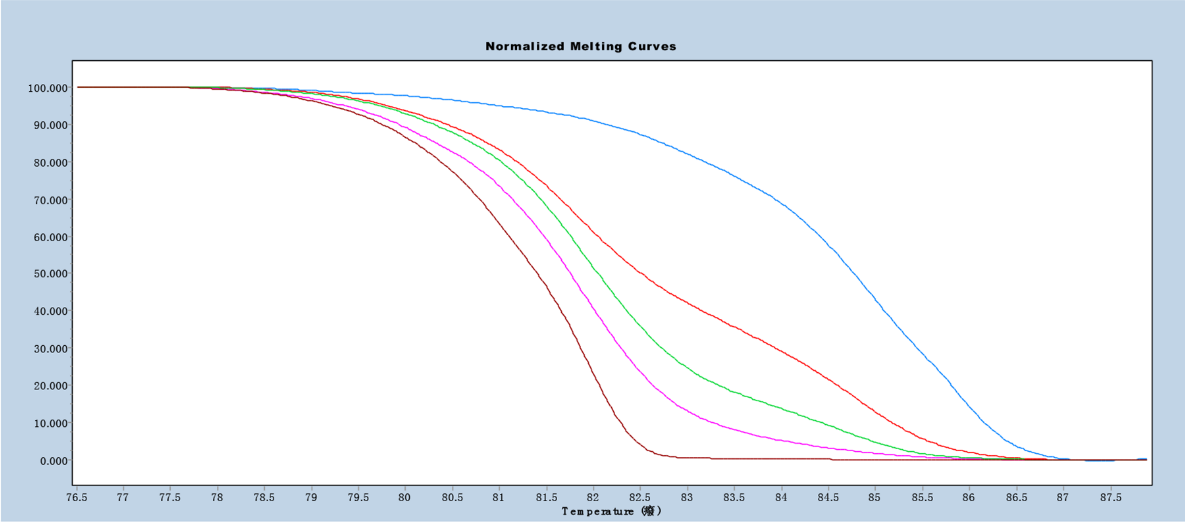
Normalized Melting Curves
100.000
90.000
80.000
70.000
60.000
50.000
40.000
30.000
20.000
10.000
0.000
76.5 77 77.5 78 78.5 79 79.5 80 80.5 81 81.5 82 82.5 83 83.5 84 84.5 85 85.5 86 86.5 87 87.5
Temperature

(*Continued*)

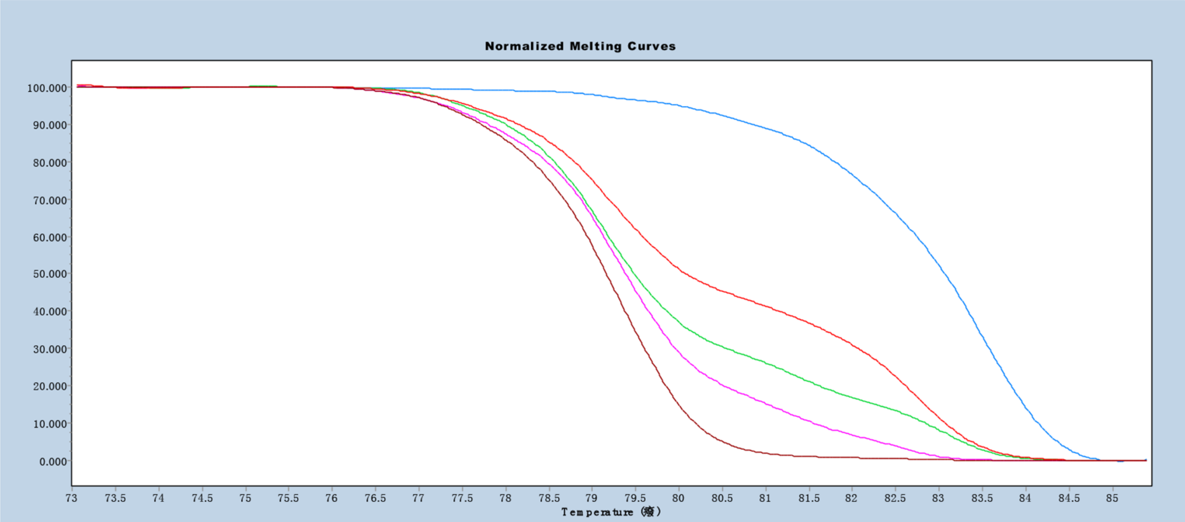
Normalized Melting Curves
100.000
90.000
80.000
70.000
60.000
50.000
40.000
30.000
20.000
10.000
0.000
73 73.5 74 74.5 75 75.5 76 76.5 77 77.5 78 78.5 79 79.5 80 80.5 81 81.5 82 82.5 83 83.5 84 84.5 85
Temperature

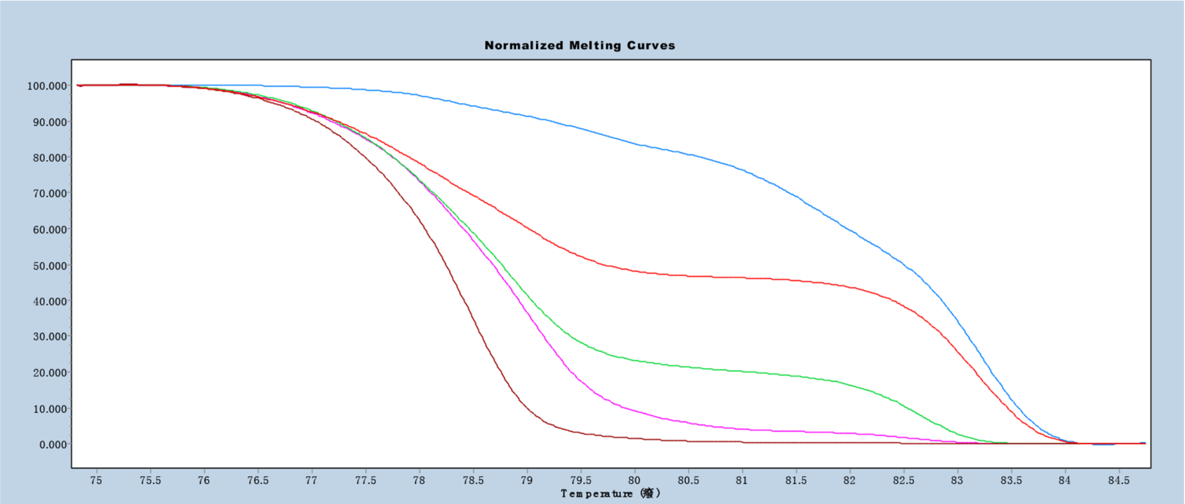
Normalized Melting Curves
100.000
90.000
80.000
70.000
60.000
50.000
40.000
30.000
20.000
10.000
0.000
75 75.5 76 76.5 77 77.5 78 78.5 79 79.5 80 80.5 81 81.5 82 82.5 83 83.5 84 84.5
Temperature

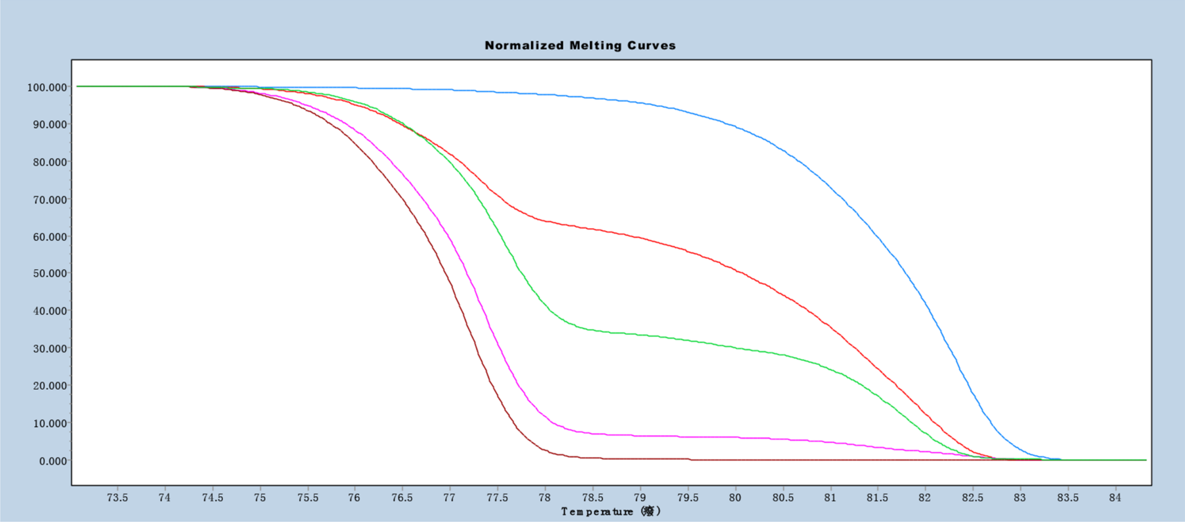
Normalized Melting Curves
100.000
90.000
80.000
70.000
60.000
50.000
40.000
30.000
20.000
10.000
0.000
73.5 74 74.5 75 75.5 76 76.5 77 77.5 78 78.5 79 79.5 80 80.5 81 81.5 82 82.5 83 83.5 84
Temperature

(*Continued*)

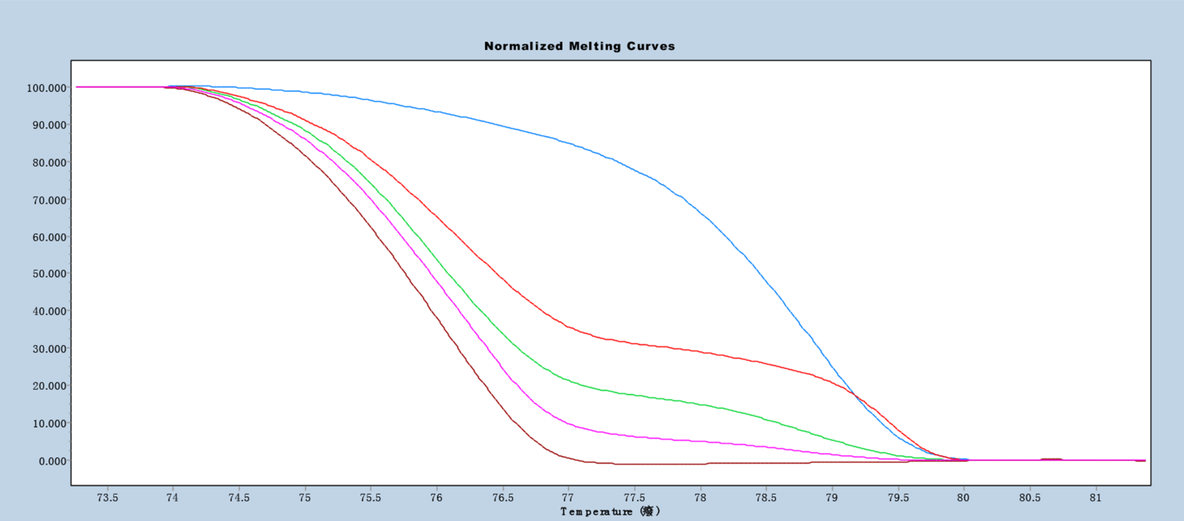
Normalized Melting Curves
100.000
90.000
80.000
70.000
60.000
50.000
40.000
30.000
20.000
10.000
0.000
73.5 74 74.5 75 75.5 76 76.5 77 77.5 78 78.5 79 79.5 80 80.5 81
Temperature

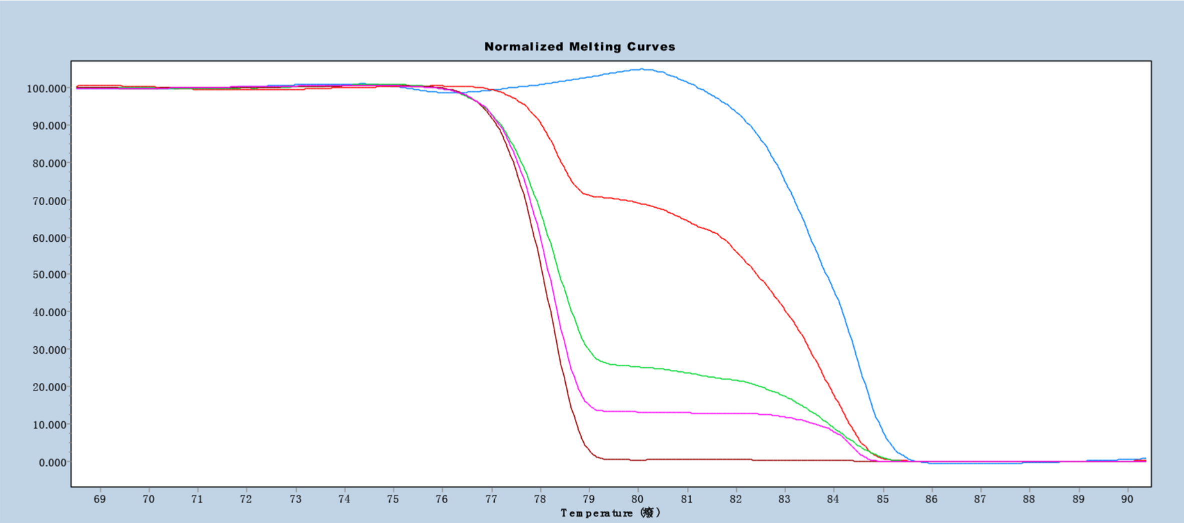
Normalized Melting Curves
100.000
90.000
80.000
70.000
60.000
50.000
40.000
30.000
20.000
10.000
0.000
69 70 71 72 73 74 75 76 77 78 79 80 81 82 83 84 85 86 87 88 89 90
Temperature

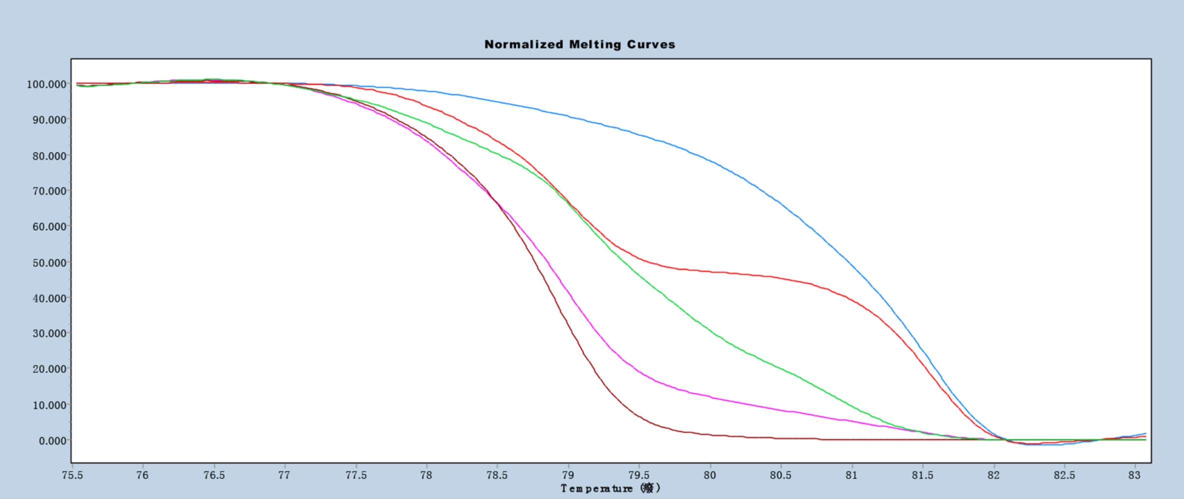
Normalized Melting Curves
100.000
90.000
80.000
70.000
60.000
50.000
40.000
30.000
20.000
10.000
0.000
75.5 76 76.5 77 77.5 78 78.5 79 79.5 80 80.5 81 81.5 82 82.5 83
Temperature

(*Continued*)

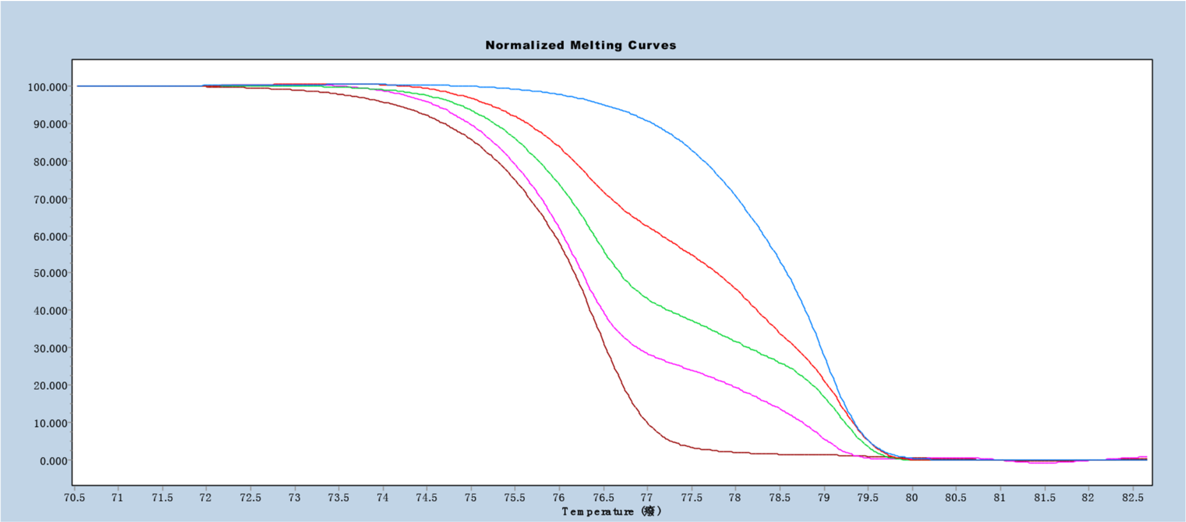

Supplementary Figure 2: Normalized melting curves of the MS-HRM assay for each gene. (A) *APC*; **(B)** *CDH1*; **(C)** *CDKN2A*; **(D)** *DAPK1*; **(E)** *IGF2*; **(F)** *MGMT*; **(G)** *MINT31*; **(H)** *MLH1*; **(I)** *NEUROG1* and **(J)** *WIF1*. Data were analyzed using the software modules of Gene Scanning. A set of dilution standards (including 100%, 5%, 2%, 1% and 0% methylated DNA) were used for all the 10 genes. The blue line refers to the normalized melting curve of the 100% methylated DNA controls, red (5%), green (2%), purple (1%) and brown (unmethylated DNA control).

Supplementary Table 1: Demographic variables and questionnaire-derived variables of participants before and after multiple imputations

See Supplementary File 1

Supplementary Table 2: Associations between DNA methylation and the risk of CRC according to gender

DNA methylation	Male						Female					
	$OR_{adjusted}^{a}$	95% CI	*P*-value	$OR_{adjusted}^{b}$	95% CI	*P*-value	$OR_{adjusted}^{a}$	95% CI	*P*-value	$OR_{adjusted}^{b}$	95% CI	*P*-value
APC	1.0079	0.3274-3.1027	0.9891	0.9307	0.2685-3.2265	0.9098	3.4501	0.8989-13.2419	0.0711	3.7099	0.8868-15.5205	0.0726
CDH1	0.5460	0.2591-1.1504	0.1115	0.5729	0.2496-1.3147	0.1887	0.6490	0.2846-1.4797	0.3039	0.7780	0.3156-1.9175	0.5854
CDKN2A	3.8048	0.7690-18.8248	0.1014	3.5483	0.6330-19.8891	0.1499	0.8452	0.1145-6.2357	0.8690	0.7466	0.0943-5.9114	0.7826
DAPK1	**3.1068**	**1.8621-5.1835**	**<0.0001**	**3.4747**	**1.9710-6.1258**	**<0.0001**	**2.6897**	**1.4684-4.9266**	**0.0014**	**2.4715**	**1.3021-4.6911**	**0.0057**
IGF2	**3.0470**	**1.7782-5.2209**	**0.0001**	**2.8244**	**1.5732-5.0709**	**0.0005**	**2.1314**	**1.1379-3.9925**	**0.0181**	**2.2493**	**1.1615-4.3558**	**0.0162**
MGMT	**1.8404**	**1.0633-3.1855**	**0.0293**	**1.8789**	**1.0329-3.4179**	**0.0388**	1.7224	0.8517-3.8431	0.1302	1.8513	0.8847-3.8742	0.1021
MINT31	**16.4418**	**2.1239-127.2812**	**0.0073**	**19.1759**	**2.3319-157.6892**	**0.0061**	1.1305	0.2924-4.3717	0.8589	1.1870	0.2882-4.8881	0.8124
MLH1	1.5214	0.5808-3.9854	0.3931	1.4222	0.5097-3.9685	0.5012	5.1908	0.5642-47.7539	0.1458	4.0104	0.3945-40.7714	0.2422
NEUROG1	**2.7349**	**1.4759-5.0681**	**0.0014**	**2.5590**	**1.3239-4.9465**	**0.0052**	**2.4343**	**1.1421-5.1884**	**0.0212**	**2.5359**	**1.1375-5.6532**	**0.0229**
WIF1	**2.4800**	**1.4249-4.3163**	**0.0013**	**2.6647**	**1.4321-4.9582**	**0.0020**	1.9645	0.9931-3.8859	0.0524	**2.2330**	**1.0881-4.5827**	**0.0285**
MRS_10-M	**4.0606**	**2.7080-6.0889**	**<0.0001**	**4.6556**	**2.9603-7.3216**	**<0.0001**	**3.0845**	**1.8228-5.2194**	**<0.0001**	**2.9376**	**1.6771-5.1454**	**<0.0001**
MRS_10-H	**9.9474**	**4.6758-21.1623**	**<0.0001**	**10.2057**	**4.4860-23.2179**	**<0.0001**	**3.8918**	**1.8773-8.0683**	**0.0003**	**4.1571**	**1.9307-8.9508**	**<0.0001**

CI: confidence interval; CRC: colorectal cancer; MRS: methylation risk score; OR: odds ratio.

[a] ORs adjusted for age and BMI.

[b] ORs adjusted for age, BMI, occupational physical activity, smoking, and consumption of coarse grains, fish stewed with brown sauce, fried food, leftovers and pork.

P values < 0.05 are in bold.

Supplementary Table 3: Associations between DNA methylation and the risk of CRC according to age

DNA methylation	<60 years						≥60 years					
	$OR_{adjusted}$[a]	95% CI	*P*-value	$OR_{adjusted}$[b]	95% CI	*P*-value	$OR_{adjusted}$[a]	95% CI	*P*-value	$OR_{adjusted}$[b]	95% CI	*P*-value
APC	1.8991	0.4762-7.5726	0.3635	1.9450	0.5108-7.4066	0.3639	1.7217	0.5895-5.0280	0.3204	1.8853	0.5669-6.2695	0.3010
CDH1	0.4675	0.1930-1.1321	0.0920	0.4518	0.1772-1.1518	0.0961	0.6847	0.3359-1.3957	0.2972	1.0010	0.4417-2.2685	0.9981
CDKN2A	5.0497	0.5747-44.3708	0.1442	6.1125	0.6072-61.5854	0.1250	1.3169	0.2835-6.1170	0.7253	0.8220	0.1605-4.2093	0.8141
DAPK1	**3.4006**	**1.8816-6.1459**	**0.0001**	**3.2927**	**1.7756-6.1059**	**0.0002**	**2.6161**	**1.5493-4.4174**	**0.0003**	**2.8258**	**1.5608-5.1160**	**0.0006**
IGF2	**2.0861**	**1.1520-3.7778**	**0.0152**	**2.1398**	**1.1412-4.0122**	**0.0177**	**3.1931**	**1.8222-5.5957**	**<0.0001**	**3.0396**	**1.6413-5.6295**	**0.0004**
MGMT	**2.3714**	**1.2580-4.4703**	**0.0076**	**2.3096**	**1.1921-4.4748**	**0.0131**	1.3931	0.7624-2.5457	0.2811	1.5256	0.7835-2.9709	0.2141
MINT31	**7.3221**	**1.5990-33.5293**	**0.0103**	**8.4610**	**1.7952-39.8787**	**0.0070**	1.9767	0.4943-7.9052	0.3353	1.7715	0.4289-7.3173	0.4294
MLH1	3.0382	0.7986-11.5590	0.1031	2.5814	0.6463-10.3109	0.1803	1.2971	0.3968-4.2407	0.6669	1.2666	0.3394-4.7259	0.7250
NEUROG1	**2.1655**	**1.0437-4.4929**	**0.0380**	2.1039	0.9806-4.5137	0.0562	**2.9836**	**1.5818-5.6275**	**0.0007**	**3.1101**	**1.5646-6.1819**	**0.0012**
WIF1	**2.2057**	**1.2066-4.0321**	**0.0102**	**2.1298**	**1.1321-4.0069**	**0.0190**	**2.3500**	**1.2724-4.3403**	**0.0063**	**2.9545**	**1.4648-5.9591**	**0.0025**
MRS_10-M	**3.3651**	**2.1487-5.2703**	**<0.0001**	**3.7130**	**2.2915-6.0163**	**<0.0001**	**4.0251**	**2.5361-6.3881**	**<0.0001**	**4.2716**	**2.5302-7.2116**	**<0.0001**
MRS_10-H	**8.8417**	**3.7590-20.7968**	**<0.0001**	**9.3211**	**3.8057-22.8295**	**<0.0001**	**5.2290**	**2.7089-10.0936**	**<0.0001**	**4.9945**	**2.4213-10.3019**	**<0.0001**

CI: confidence interval; CRC: colorectal cancer; MRS: methylation risk score; OR: odds ratio.

[a] ORs adjusted for gender and BMI.

[b] ORs adjusted for gender, BMI, occupational physical activity, smoking, and consumption of coarse grains, fish stewed with brown sauce, fried food, leftovers and pork.

P values < 0.05 are in bold.

Supplementary Table 4: Associations between DNA methylation and the risk of CRC according to body mass index

DNA methylation	<24						≥24					
	$OR_{adjusted}$ [a]	95% CI	*P*-value	$OR_{adjusted}$ [b]	95% CI	*P*-value	$OR_{adjusted}$ [a]	95% CI	*P*-value	$OR_{adjusted}$ [b]	95% CI	*P*-value
APC	1.2586	0.4141-3.8258	0.6851	1.3584	0.4184-4.4103	0.6103	2.6796	0.7625-9.4162	0.1243	3.0226	0.7612-12.0024	0.1159
CDH1	0.8938	0.4304-1.8561	0.7634	1.0214	0.4619-2.2583	0.9584	**0.3038**	**0.1128-0.8184**	**0.0185**	**0.2903**	**0.0969-0.8696**	**0.0271**
CDKN2A	4.2718	0.5095-35.8148	0.1808	4.2958	0.4804-38.4095	0.1948	1.4362	0.2858-7.2177	0.6603	1.2506	0.2135-7.3274	0.8042
DAPK1	**3.1913**	**1.7938-5.6776**	**0.0001**	**3.5033**	**1.8923-6.4859**	**0.0001**	**2.7381**	**1.6013-4.6819**	**0.0002**	**2.5878**	**1.4305-4.6816**	**0.0017**
IGF2	**2.7907**	**1.5962-4.8789**	**0.0003**	**2.7255**	**1.5170-4.8967**	**0.0008**	**2.4530**	**1.3469-4.4673**	**0.0033**	**2.3984**	**1.2345-4.6596**	**0.0098**
MGMT	1.6104	0.8450-3.0694	0.1476	1.6306	0.8301-3.2031	0.1558	**1.9454**	**1.0933-3.4616**	**0.0236**	**1.9636**	**1.0368-3.7189**	**0.0384**
MINT31	2.1544	0.5734-8.0942	0.2557	2.7438	0.7069-10.6498	0.1447	**7.3458**	**1.5821-34.1059**	**0.0109**	**8.1506**	**1.6438-40.4154**	**0.0102**
MLH1	1.7643	0.5397-5.7621	0.3477	1.7262	0.4877-6.1090	0.3973	2.1464	0.5965-7.7226	0.2423	1.5976	0.3991-6.3953	0.5081
NEUROG1	**2.6286**	**1.3289-5.1993**	**0.0055**	**2.9123**	**1.4306-5.9287**	**0.0032**	**2.5921**	**1.3240-5.0747**	**0.0055**	**2.1920**	**1.0520-4.5675**	**0.0361**
WIF1	**2.2053**	**1.1815-4.1163**	**0.0130**	**2.7322**	**1.3958-5.3483**	**0.0034**	**2.3365**	**1.2928-4.2228**	**0.0049**	**2.2652**	**1.1726-4.3757**	**0.0149**
MRS_10-M	**3.7159**	**2.3484-5.8797**	**<0.0001**	**4.5709**	**2.7525-7.5908**	**<0.0001**	**3.6523**	**2.3235-5.7410**	**<0.0001**	**3.4009**	**2.0641-5.6035**	**<0.0001**
MRS_10-H	**6.0499**	**2.9690-12.3281**	**<0.0001**	**6.7389**	**3.1595-14.3733**	**<0.0001**	**6.7316**	**3.2076-14.1274**	**<0.0001**	**6.6814**	**2.9886-14.9370**	**<0.0001**

CI: confidence interval; CRC: colorectal cancer; MRS: methylation risk score; OR: odds ratio.

[a] ORs adjusted for age, gender and BMI.

[b] ORs adjusted for gender, BMI, occupational physical activity, smoking, and consumption of coarse grains, fish stewed with brown sauce, fried food, leftovers and pork.

P values < 0.05 are in bold.

Supplementary Table 5: Reclassification table of individuals of predicted risk using the combined EF and MRS_10 model versus the EF-only model

**Basic model**	**Combined EF and MRS_10 model**		
EF-only model	≤0.5	>0.5	RC%
≤0.5			
Total	358	77	18
Case	97	57	37
Control	261	20	7
>0.5			
Total	88	333	21
Case	31	243	11
Control	57	90	39
NRI (95% CI)	**0.1472 (0.0892-0.2052), *P*<0.0001**		
IDI (95% CI)	**0.0930 (0.0736-0.1123), *P*<0.0001**		
Delta-AUC (95% CI)	**0.0618 (0.0399-0.0854), *P*<0.0001**		

AUC: area under the curve; CI: confidence interval; Delta-AUC: the difference of AUCs between the basic model and the extended model with the MRS; EF: environmental factors; IDI: integrated discrimination improvement; MRS: methylation risk score; NRI: net reclassification improvement; RC%: reclassified percent (%).
P values < 0.05 are in bold.

Supplementary Table 6: Relationship between environmental factors and DNA methylation

See Supplememtary File 1

Supplementary Table 7: Sensitivity analysis for the associations between MRS_10 and CRC risk by omitting each gene at a time

See Supplememtary File 1

Supplementary Table 8: Correlation between DNA methylation levels in leukocytes and matched colorectal tumour tissues in patients with CRC

Gene	*APC*	*CDKN2A*	*MGMT*	*APBA1*	*MINT31*	*MLH1*
Number of Cases	211	213	211	205	204	205
Spearman's Rho	-0.1487	0.0080	0.0267	0.0695	0.0914	0.0003
P-value	0.0301	0.9079	0.6995	0.3218	0.1934	0.9967

Supplementary Table 9: Sensitivity analysis for the categorical net reclassification improvement at different cut-off points

Basic Model	NRI at Different Cut-Off Points	Combined EF and MRS_10
EF-Only	$NRI^{0.3}$	**0.0911 (0.0437-0.1385), *P*=0.0002**
	$NRI^{0.4}$	**0.0841 (0.0259-0.1423), *P*=0.0046**
	$NRI^{0.6}$	**0.1238 (0.0633-0.1844), *P*<0.0001**
	$NRI^{0.7}$	**0.1168 (0.0615-0.1721), *P*<0.0001**

AUC: area under the curve; CI: confidence interval; EF: environmental factors; MRS: methylation risk score; NRI: net reclassification improvement.
P values < 0.05 are in bold.

Supplementary Table 10: Sequences of primer sets used for methylation-sensitive high-resolution melting analysis

See Supplememtary File 1

Supplementary Table 11: Sequences of primer sets used for pyrosequencing

Gene		Primer sequences: 5'-3'	CpG Sites/ Amplicon size (bp)	Annealing temperature (°C)
DAPK1	Forward PCR primer	GCGCGGAGTTGGGAGGAGT	7/70	56
	Reverse PCR primer	Biotin-CTCCGAACTACCCTACCAAACC		
	Sequencing primer	GCGCGGAGTTGGGAGGAGT		
	Analyzed sequence	AGYGAGYGTYGYGTAGAATTYGTAGYGTYGGTTTGGTAGGGTAGTT		
	Dispensation order	GATGTCGATGTCAGTCAGTCGTAGTATCGTATGTCAGTCGTG		
MLH1[a]	Forward PCR primer	GGAGTGAAGGAGGTTACGGGTAAGT	22/182	56
	Reverse PCR primer	Biotin-AAAAACGATAAAACCCTATACCTAATCTATC		
	Sequencing primer_1	GGAGTGAAGGAGGTTACGGGTAAGT		
	Analyzed sequence_1	YGGGTAAGTYGTTTTGAYGTAGAYGTTTTATTAGGGTYGYGYGTTYGTYGTTYGTTATATATYGTTYGTAGTATTYGTGTTTAGTTTYGTAGTGG		
	Dispensation order_1	ATCGTATGTCTGTTGTATCGTAGTATCGTTATATGTCTAGTCAGTCAGTCTAGTCTAGTCTGTATATGATCTAGTCTGTAGTGATCTGTGTATGTTCTGTA		
	Sequencing primer_2	GTAGTATTAGTGTTTAGTTT		
	Analyzed sequence_2	YGTAGTGGYGTTTGAYGTYGYGTTYGYGGGTAGTTAYGATGAGGYGGYGATAGATTAGGTATAGGGTTTTAT		
	Dispensation order_2	GTCGTAGTAGTCGTGTATCAGTCTAGTCAGTCTAGTCGTAGTGATCGATGATGTCAGTCGAT		

[a] For *MLH1*, the size of the PyroMark PCR-amplified DNA template is 182bp, which is too long for pyrosequencing in a single pyrosequencing reaction; thus, we performed two pyrosequencing reaction using two sets of sequencing primers.

Supplementary Table 12: Estimates of regression coefficients and odds ratios from the models[a]

See Supplememtary File 1
