# Supplementary material for: Multiple gene-specific DNA methylation in blood leukocytes and colorectal cancer risk: a case-control study in China
Source: Oncotarget. 2017 May 22;8(37):61239–52. doi: 10.18632/oncotarget.18054 (PMC5617420; doi:10.18632/oncotarget.18054)
Supplement: Supplementary file 2 [file oncotarget-08-61239-s002.doc]

Supplementary Table 1: Demographic variables and questionnaire-derived variables of participants before and after multiple imputations

| Characteristics |  | Entire Data Set |
| --- | --- | --- |
|  |
|  | Pre-imputation | Post-imputation |
|  | No. (%) | No. (%) |
| Age, years |  |  |  |
| Mean (SD) |  | 59.36 (10.32) | NA |
| Median (IQR) |  | 59.00 (53.00-67.00) |
| Gender |  |  |  |
| Male |  | 532 (62.15) | NA |
| Female |  | 324 (37.85) |
| BMI, kg/m2 |  |  |  |
| Mean (SD) |  | 25.59 (7.52) | NA |
| Median (IQR) |  | 24.03 (21.49-26.78) |
| Coarse grains |  |  |  |
| <50 g/week |  | 342 (39.95) | 342 (39.95) |
| ≥50 g/week |  | 512 (59.81) | 514 (60.05) |
| Missing |  | 2 (0.23) | - |
| Dairy |  |  |  |
| <1 time/week |  | 366 (42.78) | 470 (54.91) |
| ≥1 time/week |  | 284 (33.18) | 386 (45.09) |
| Missing |  | 206 (24.07) | - |
| Family history of any cancers other than CRC in first-degree relatives |  |  |  |
| Yes |  | 126 (14.72) | 130 (15.19) |
| No |  | 706 (82.48) | 726 (84.81) |
| Missing |  | 24 (2.80) | - |
| Fish stewed with brown sauce |  |  |  |
| <1 time/week |  | 595 (69.51) | 604 (70.56) |
| ≥1 time/week |  | 250 (29.21) | 252 (29.44) |
| Missing |  | 11 (1.28) | - |
| Fried food |  |  |  |
| <1 time/month |  | 635 (74.18) | 638 (74.53) |
| ≥1 time/month |  | 216 (25.23) | 218 (25.47) |
| Missing |  | 5 (0.58) | - |
| Fresh fruits |  |  |  |
| <2 times/week |  | 404 (47.20) | 437 (51.05) |
| ≥2 times/week |  | 402 (46.96) | 419 (49.95) |
| Missing |  | 50 (5.84) | - |
| Green vegetables |  |  |  |
| <100 g/day |  | 68 (7.94) | 75 (8.76) |
| ≥100 g/day |  | 736 (85.98) | 781 (91.24) |
| Missing |  | 52 (6.07) | - |
| Occupational physical activity |  |  |  |
| Blue-collar worker |  | 542 (63.32) | 553 (64.60) |
| White-collar worker |  | 301 (35.16) | 303 (35.40) |
| Missing |  | 13 (1.52) | - |
| Leftover |  |  |  |
| <1 time/week |  | 533 (62.27) | 538 (62.85) |
| ≥1 time/week |  | 316 (36.91) | 318 (37.15) |
| Missing |  | 7 (0.82) | - |
| Pork |  |  |  |
| <250 g/week |  | 463 (54.09) | 476 (55.61) |
| ≥250 g/week |  | 369 (43.11) | 380 (44.39) |
| Missing |  | 24 (2.80) | - |
| Smoking |  |  |  |
| No |  | 460 (53.74) | NA |
| Yes |  | 396 (46.26) |
| Soybean |  |  |  |
| <1 time/week |  | 548 (64.02) | 548 (64.14) |
| ≥1 time/week |  | 307 (35.86) | 308 (35.86) |
| Missing |  | 1 (0.12) | - |

BMI: body mass index; IQR: inter-quartile range; NA: not applicable; SD: standard deviation.

Supplementary Table 6: Relationship between environmental factors and DNA methylation

| Environmental Factor | MRS_10 |  | DAPK1 |  | NEUROG1 |  |  |  |  |  | CDH1 |  |  |  |
| --- | --- | --- | --- | --- | --- | --- | --- | --- | --- | --- | --- | --- | --- | --- |
| All subjects |  | Cases |  | Controls |  | All subjects |  | Cases |  | Controls |  | All subjects |  | Cases |  | Controls |  | All subjects |  | Cases |  | Controls |
| OR (95% CI)a | P |  | OR (95% CI)b | P |  | OR (95% CI)b | P |  | OR (95% CI)a | P |  | OR (95% CI)b | P |  | OR (95% CI)b | P |  | OR (95% CI)a | P |  | OR (95% CI)b | P |  | OR (95% CI)b | P |  | OR (95% CI)a | P |  | OR (95% CI)b | P |  | OR (95% CI)b | P |
| Smoking | No (ref) | 1.00 |  |  | 1.00 |  |  | 1.00 |  |  | 1.00 |  |  | 1.00 |  |  | 1.00 |  |  | 1.00 |  |  | 1.00 |  |  | 1.00 |  |  | 1.00 |  |  | 1.00 |  |  | 1.00 |  |
| Yes | 1.48 (1.08-2.04) | 0.0151 |  | 1.77 (1.15-2.72) | 0.0096 |  | 1.21 (0.75-1.95) | 0.4351 |  | 1.55 (1.05-2.30) | 0.0288 |  | 1.62 (1.00-2.62) | 0.0490 |  | 1.45 (0.72-2.89) | 0.2955 |  | 1.37 (0.85-2.20) | 0.1918 |  | 1.61 (0.91-2.85) | 0.1031 |  | 0.98 (0.42-2.29) | 0.9619 |  | 1.36 (0.76-2.42) | 0.2955 |  | 0.81 (0.32-2.09) | 0.6689 |  | 2.05 (0.95-4.41) | 0.0664 |
| Pork (g/week) | <250 (ref) | 1.00 |  |  | 1.00 |  |  | 1.00 |  |  | 1.00 |  |  | 1.00 |  |  | 1.00 |  |  | 1.00 |  |  | 1.00 |  |  | 1.00 |  |  | 1.00 |  |  | 1.00 |  |  | 1.00 |  |
| ≥250 | 1.19 (0.88-1.62) | 0.2683 |  | 1.22 (0.81-1.86) | 0.3379 |  | 1.14 (0.72-1.82) | 0.5774 |  | 1.33 (0.91-1.94) | 0.1388 |  | 1.31 (0.83-2.08) | 0.2506 |  | 1.37 (0.71-2.65) | 0.3517 |  | 1.76 (1.11-2.80) | 0.0167 |  | 1.30 (0.75-2.27) | 0.3525 |  | 3.57 (1.50-8.49) | 0.0041 |  | 1.16 (0.66-2.04) | 0.5981 |  | 2.04 (0.68-6.09) | 0.2026 |  | 1.45 (0.70-3.00) | 0.3158 |
| Leftover (times/week) | <1 (ref) | 1.00 |  |  | 1.00 |  |  | 1.00 |  |  | 1.00 |  |  | 1.00 |  |  | 1.00 |  |  | 1.00 |  |  | 1.00 |  |  | 1.00 |  |  | 1.00 |  |  | 1.00 |  |  | 1.00 |  |
| ≥1 | 1.22 (0.90-1.64) | 0.2004 |  | 1.41 (0.94-2.11) | 0.0942 |  | 1.01 (0.64-1.62) | 0.9555 |  | 1.50 (1.04-2.17) | 0.0293 |  | 1.54 (0.98-2.40) | 0.0597 |  | 1.48 (0.77-2.83) | 0.2372 |  | 1.28 (0.82-2.00) | 0.2835 |  | 1.21 (0.71-2.08) | 0.4846 |  | 1.45 (0.64-3.26) | 0.3721 |  | 0.81 (0.45-1.45) | 0.4788 |  | 0.52 (0.20-1.36) | 0.1843 |  | 1.19 (0.56-2.53) | 0.6511 |
| Fish stewed with brown sauce (times/week) | <1 (ref) | 1.00 |  |  | 1.00 |  |  | 1.00 |  |  | 1.00 |  |  | 1.00 |  |  | 1.00 |  |  | 1.00 |  |  | 1.00 |  |  | 1.00 |  |  | 1.00 |  |  | 1.00 |  |  | 1.00 |  |
| ≥1 | 1.05 (0.76-1.44) | 0.7814 |  | 1.29 (0.85-1.94) | 0.2312 |  | 0.75 (0.44-1.30) | 0.3065 |  | 1.09 (0.74-1.61) | 0.6791 |  | 1.16 (0.70-1.77) | 0.6424 |  | 1.02 (0.48-2.17) | 0.9557 |  | 0.88 (0.54-1.43) | 0.6092 |  | 0.72 (0.41-1.29) | 0.2748 |  | 0.72 (0.41-1.29) | 0.2748 |  | 1.55 (0.88-2.74) | 0.1308 |  | 3.28 (1.36-7.91) | 0.0082 |  | 0.82 (0.35-1.94) | 0.6477 |

CI: confidence interval; MRS: methylation risk score; OR: odds ratio.

a ORs adjusted for age, gender, BMI and case-control status.

b ORs adjusted for age, gender and BMI.

P values < 0.05 are in bold.

Supplementary Table 7: Sensitivity analysis for the associations between MRS_10 and CRC risk by omitting each gene at a time

| DNA Methylation Status |  | Crude OR | 95% CI | P | ORadjusteda | 95% CI | P | ORadjustedb | 95% CI | P |
| --- | --- | --- | --- | --- | --- | --- | --- | --- | --- | --- |
| MRS_9 |  |  |  |  |  |  |  |  |  |  |  |
|  | APC | Low | 1.00 |  |  | 1.00 |  |  | 1.00 |  |  |
|  |  | Medium | 3.62 | 2.63-4.99 | <0.0001 | 3.51 | 2.53-4.86 | <0.0001 | 3.77 | 2.63-5.42 | <0.0001 |
|  |  | High | 6.45 | 3.99-10.44 | <0.0001 | 6.24 | 3.84-10.16 | <0.0001 | 5.93 | 3.48-10.09 | <0.0001 |
|  |  | Medium or High | 4.27 | 3.20-5.71 | <0.0001 | 4.13 | 3.08-5.55 | <0.0001 | 4.29 | 3.09-5.96 | <0.0001 |
|  | CDH1 | Low | 1.00 |  |  | 1.00 |  |  | 1.00 |  |  |
|  |  | Medium | 3.47 | 2.55-4.74 | <0.0001 | 3.39 | 2.48-4.64 | <0.0001 | 3.63 | 2.56-5.16 | <0.0001 |
|  |  | High | 5.61 | 3.49-9.03 | <0.0001 | 5.36 | 3.31-8.69 | <0.0001 | 5.51 | 3.23-9.40 | <0.0001 |
|  |  | Medium or High | 3.93 | 2.96-5.23 | <0.0001 | 3.81 | 2.85-5.09 | <0.0001 | 4.04 | 2.92-5.59 | <0.0001 |
|  | CDKN2A | Low | 1.00 |  |  | 1.00 |  |  | 1.00 |  |  |
|  |  | Medium | 3.57 | 2.60-4.91 | <0.0001 | 3.46 | 2.51-4.78 | <0.0001 | 3.66 | 2.55-5.25 | <0.0001 |
|  |  | High | 7.07 | 4.27-11.69 | <0.0001 | 6.80 | 4.09-11.31 | <0.0001 | 6.57 | 3.77-11.45 | <0.0001 |
|  |  | Medium or High | 4.28 | 3.20-5.73 | <0.0001 | 4.14 | 3.08-5.57 | <0.0001 | 4.27 | 3.07-5.95 | <0.0001 |
|  | DAPK1 | Low | 1.00 |  |  | 1.00 |  |  | 1.00 |  |  |
|  |  | Medium | 3.18 | 2.30-4.39 | <0.0001 | 3.00 | 2.17-4.17 | <0.0001 | 2.95 | 2.06-4.24 | <0.0001 |
|  |  | High | 4.54 | 2.88-7.15 | <0.0001 | 4.47 | 2.82-7.09 | <0.0001 | 4.50 | 2.72-7.46 | <0.0001 |
|  |  | Medium or High | 3.54 | 2.66-4.73 | <0.0001 | 3.40 | 2.54-4.55 | <0.0001 | 3.36 | 2.43-4.64 | <0.0001 |
|  | IGF2 | Low | 1.00 |  |  | 1.00 |  |  | 1.00 |  |  |
|  |  | Medium | 3.08 | 2.19-4.33 | <0.0001 | 3.06 | 2.16-4.32 | <0.0001 | 3.30 | 2.24-4.84 | <0.0001 |
|  |  | High | 6.36 | 3.82-10.56 | <0.0001 | 6.27 | 3.75-10.48 | <0.0001 | 5.84 | 3.35-10.18 | <0.0001 |
|  |  | Medium or High | 3.86 | 2.86-5.22 | <0.0001 | 3.83 | 2.82-5.19 | <0.0001 | 3.94 | 2.80-5.54 | <0.0001 |
|  | MGMT | Low | 1.00 |  |  | 1.00 |  |  | 1.00 |  |  |
|  |  | Medium | 3.58 | 2.60-4.93 | <0.0001 | 3.47 | 2.51-4.80 | <0.0001 | 3.73 | 2.60-5.35 | <0.0001 |
|  |  | High | 6.72 | 4.13-10.95 | <0.0001 | 6.48 | 3.96-10.60 | <0.0001 | 6.15 | 3.60-10.52 | <0.0001 |
|  |  | Medium or High | 4.27 | 3.20-5.71 | <0.0001 | 4.13 | 3.08-5.55 | <0.0001 | 4.29 | 3.09-5.96 | <0.0001 |
|  | MINT31 | Low | 1.00 |  |  | 1.00 |  |  | 1.00 |  |  |
|  |  | Medium | 3.51 | 2.55-4.82 | <0.0001 | 3.35 | 2.42-4.63 | <0.0001 | 3.53 | 2.46-5.06 | <0.0001 |
|  |  | High | 6.25 | 3.80-10.27 | <0.0001 | 6.20 | 3.74-10.27 | <0.0001 | 5.46 | 3.18-9.38 | <0.0001 |
|  |  | Medium or High | 4.09 | 3.06-5.48 | <0.0001 | 3.95 | 2.94-5.31 | <0.0001 | 3.98 | 2.86-5.53 | <0.0001 |
|  | MLH1 | Low | 1.00 |  |  | 1.00 |  |  | 1.00 |  |  |
|  |  | Medium | 3.37 | 2.45-4.65 | <0.0001 | 3.27 | 2.37-4.53 | <0.0001 | 3.44 | 2.39-4.93 | <0.0001 |
|  |  | High | 6.72 | 4.06-11.11 | <0.0001 | 6.51 | 3.91-10.83 | <0.0001 | 6.24 | 3.58-10.87 | <0.0001 |
|  |  | Medium or High | 4.07 | 3.04-5.44 | <0.0001 | 3.94 | 2.94-5.30 | <0.0001 | 4.03 | 2.90-5.61 | <0.0001 |
|  | NEUROG1 | Low | 1.00 |  |  | 1.00 |  |  | 1.00 |  |  |
|  |  | Medium | 3.31 | 2.37-4.62 | <0.0001 | 3.17 | 2.26-4.45 | <0.0001 | 3.25 | 2.23-4.74 | <0.0001 |
|  |  | High | 6.04 | 3.72-9.80 | <0.0001 | 5.91 | 3.63-9.65 | <0.0001 | 5.41 | 3.17-9.21 | <0.0001 |
|  |  | Medium or High | 3.99 | 2.97-5.38 | <0.0001 | 3.86 | 2.86-5.21 | <0.0001 | 3.82 | 2.73-5.33 | <0.0001 |
|  | WIF1 | Low | 1.00 |  |  | 1.00 |  |  | 1.00 |  |  |
|  |  | Medium | 2.80 | 2.05-3.82 | <0.0001 | 2.73 | 1.99-3.75 | <0.0001 | 2.53 | 1.78-3.60 | <0.0001 |
|  |  | High | 7.01 | 4.29-11.48 | <0.0001 | 6.85 | 4.17-11.28 | <0.0001 | 6.41 | 3.76-10.93 | <0.0001 |
|  |  | Medium or High | 3.60 | 2.71-4.79 | <0.0001 | 3.51 | 2.63-4.69 | <0.0001 | 3.29 | 2.39-4.52 | <0.0001 |

CI: confidence interval; CRC: colorectal cancer; MRS: methylation risk score; OR: odds ratio.

a ORs adjusted for age, gender and BMI.

b ORs adjusted for age, gender, BMI, occupational physical activity, smoking, and consumption of coarse grains, fish stewed with brown sauce, fried food, leftovers and pork.

P values < 0.05 are in bold.

Supplementary Table 10: Sequences of primer sets used for methylation-sensitive high-resolution melting analysis

| Gene | Primer Sequence (5'-3') | GenBank Accession | Position/Genomic Region | Sense/Antisense Complementary | CpG Sites/Amplicon Size (bp) | CpG Islanda | Annealing Temperature (℃) | Final Concentration of Each Primer (µM) |
| --- | --- | --- | --- | --- | --- | --- | --- | --- |
| APC51 | F-AAGTAGTTGTGTAATTCGTTGGAT | NG_008481.4 | chr5:112073454 -112073602 | S | 10/149 | Y | 57 | 0.2 |
| R-CACCTCCATTCTATCTCCAAT |
| CDH152 | F-GAGTTTGCGGAAGTTAGTTTAGATTTTAG | NG_008021.1 | chr16:68771223 -68771335 | S | 11/113 | N | 58 | 0.2 |
| R-CGACTCCAAAAACCCATAACTAACC |
| CDKN2A53 | F-GGAGTTTTCGGTTGATTGGTTGGTT | NG_007485.1 | chr9:21970080 -21970151 | S | 5/69 | Y | 57 | 0.2 |
| R-AACAACGCCCGCACCTCCTCTA |
| CDKN2B54 | F-GTTAGGCGTTTTTTTTTAGAAGTAATTTAGG | NG_023297.1 | chr9:22004998 -22005111 | S | 9/114 | Y | 58 | 0.2 |
| R-TACGACTTAAAACCCCGTACAATAACC |
| DAPK155 | F-GCGCGGAGTTGGGAGGAGT | NM_004938.3 | chr9:87497703 -87497702 | S | 7/70 | Y | 65 | 0.3 |
| R-CTCCGAACTACCCTACCAAACC |
| GSTP156 | F-GTGAAGCGGGTGTGTAAGTTT | NG_012075.1 | chr11:67350923 -67351042 | S | 12/120 | N | 58 | 0.2 |
| R-TAAACAAACAACAAAAAAAAAACC |
| IGF2 | F-GGGATTTGGTTGAGGTTTTAAG | NG_008849.1 | chr11:2125408 -2125508 | S | 9/101 | Y | 56 | 0.4 |
| R-TACGACTAAAAAAACCCCTAAACTC |
| MGMT57 | F-GCGTTTCGGATATGTTGGGATAGT | X61657.1 | chr10:131155459 -131155568 | S | 12/110 | N | 58 | 0.2 |
| R-AACGACCCAAACACTCACCAAA |
| APBA1 | F-GGGGTTGAGGTTTTTTGTTAG | AF135501.1 | chr5:76084084 -76084220 | S | 7/137 | Y | 59 | 0.2 |
| R-AATCCCTCTCCCCTCTAAACTT |
| MINT31 | F-GGGTGATGGTTTTAGTAAAGTGAG | AF135531.1 | chr17:50558315 -50558479 | S | 10/164 | Y | 56 | 0.2 |
| R-AAAAACACTTCCCCAACATCTAC |
| MLH1b | F-GGAGTGAAGGAGGTTACGGGTAAGT | U83845.1 | chr3:37034291 -37034472 | S | 22/182 | Y | 52 | 0.4 |
| R-AAAAACGATAAAACCCTATACCTAATCTATC |
| NEUROG1 | F-AGCGGAGGTTGTTTGTTGGAG | AC005738 | chr5:135536548 -135536663 | AC | 7/116 | Y | 58 | 0.2 |
| R-CCCGCCTTAAAACCTACATCT |
| PTEN56 | F-TCGGTTGGGTTTTTGGGTAGAGG | AF406618.1 | chr10:89622968 -89623124 | S | 9/156 | N | 56 | 0.2 |
| R-CGCAAACTCTACTAAACATACCCAATAT |
| WIF1 | F-GAGGTGGCGAGTGATGTTTTAGG | AC026124.35 | chr12:65446217 -65446337 | S | 8/121 | Y | 58 | 0.2 |
| R-AACCCCCGAAACTACATTCACAATA |

AC: antisense complementary strand; S: sense strand.

a CpG islands were predicted using MethPrimer (http://www.urogene.org/methprimer/).

b Provided by Zymo Research Company.

Supplementary Table 12: Estimates of regression coefficients and odds ratios from the modelsa

| Factors | Status | EF-Only Model |  | MRS_10 Model |  | Combined EF and MRS_10 Model |
| --- | --- | --- | --- | --- | --- | --- |
| β-coefficient | P-value | OR | 95% CI |  | β-coefficient | P-value | OR | 95% CI |  | β-coefficient | P-value | OR | 95% CI |
| Lower | Upper |  | Lower | Upper |  | Lower | Upper |
| Constant |  | 0.0359 |  |  |  |  |  | -0.5443 |  |  |  |  |  | -0.4587 |  |  |  |  |
| Age (years) | <60 (ref) |  |  |  |  |  |  | - | - | - | - | - |  |  |  |  |  |  |
| ≥60 | -0.0898 | 0.5572 | 0.9141 | 0.6773 | 1.2337 |  | - | - | - | - | - |  | -0.1856 | 0.2531 | 0.8306 | 0.6041 | 1.1419 |
| Gender | Male (ref) |  |  |  |  |  |  | - | - | - | - | - |  |  |  |  |  |  |
| Female | 0.1071 | 0.5294 | 1.1131 | 0.7971 | 1.5543 |  | - | - | - | - | - |  | 0.0420 | 0.8164 | 1.0428 | 0.7319 | 1.4860 |
| BMI (kg/m2) | <24 (ref) |  |  |  |  |  |  | - | - | - | - | - |  |  |  |  |  |  |
| ≥24 | -0.8521 | <0.0001 | 0.4265 | 0.3165 | 0.5749 |  | - | - | - | - | - |  | -0.7577 | <0.0001 | 0.4688 | 0.3416 | 0.6432 |
| Coarse grains | <50 (ref) |  |  |  |  |  | - | - | - | - | - |  |  |  |  |  |  |
| (g/week) | ≥50 | -0.7768 | <0.0001 | 0.4599 | 0.3385 | 0.6274 |  | - | - | - | - | - |  | -0.7916 | <0.0001 | 0.4531 | 0.3270 | 0.6279 |
| Fish stewed with brown sauce (time/week) | <1 (ref) |  |  |  |  |  | - | - | - | - | - |  |  |  |  |  |  |
| ≥1 | 0.5277 | 0.0016 | 1.6950 | 1.2216 | 2.3519 |  | - | - | - | - | - |  | 0.5330 | 0.0026 | 1.7040 | 1.2043 | 2.4110 |
| Fried food (time/month) | <1 (ref) |  |  |  |  |  | - | - | - | - | - |  |  |  |  |  |  |
| ≥1 | 0.6488 | 0.0002 | 1.9133 | 1.3550 | 2.7016 |  | - | - | - | - | - |  | 0.6002 | 0.0012 | 1.8225 | 1.2659 | 2.6238 |
| Occupational physical activity | Blue-collar (ref) |  |  |  |  |  | - | - | - | - | - |  |  |  |  |  |  |
| White-collar | 1.1194 | <0.0001 | 3.0629 | 2.2197 | 4.2264 |  | - | - | - | - | - |  | 1.1836 | <0.0001 | 3.2660 | 2.3080 | 4.6018 |
| Leftover (time/week) | <1 (ref) |  |  |  |  |  | - | - | - | - | - |  |  |  |  |  |  |
| ≥1 | 0.4067 | 0.0089 | 1.5032 | 1.1078 | 2.0398 |  | - | - | - | - | - |  | 0.3466 | 0.0360 | 1.4142 | 1.0228 | 1.9553 |
| Pork (g/week) | <250 (ref) |  |  |  |  |  | - | - | - | - | - |  |  |  |  |  |  |
| ≥250 | 0.3564 | 0.0258 | 1.4283 | 1.0440 | 1.9539 |  | - | - | - | - | - |  | 0.2947 | 0.0879 | 1.3428 | 0.9571 | 1.8838 |
| Smoking | No (ref) |  |  |  |  |  |  | - | - | - | - | - |  |  |  |  |  |  |
| Yes | 0.3269 | 0.0443 | 1.3866 | 1.0084 | 1.9069 |  | - | - | - | - | - |  | -0.4531 | 0.0089 | 1.5732 | 1.1204 | 2.2090 |
| APC | No (ref) | - | - | - | - | - |  |  |  |  |  |  |  | - | - | - | - | - |
| Yes | - | - | - | - | - |  | 0.3460 | 0.4518 | 1.4134 | 0.5740 | 3.4804 |  | - | - | - | - | - |
| CDH1 | No (ref) | - | - | - | - | - |  |  |  |  |  |  |  | - | - | - | - | - |
| Yes | - | - | - | - | - |  | -1.0498 | 0.0008 | 0.3500 | 0.1898 | 0.6456 |  | - | - | - | - | - |
| CDKN2A | No (ref) | - | - | - | - | - |  |  |  |  |  |  |  | - | - | - | - | - |
| Yes | - | - | - | - | - |  | 1.0342 | 0.1178 | 2.8128 | 0.7696 | 10.2804 |  | - | - | - | - | - |
| DAPK1 | No (ref) | - | - | - | - | - |  |  |  |  |  |  |  | - | - | - | - | - |
| Yes | - | - | - | - | - |  | 0.9756 | <0.0001 | 2.6526 | 1.7705 | 3.9743 |  | - | - | - | - | - |
| IGF2 | No (ref) | - | - | - | - | - |  |  |  |  |  |  |  | - | - | - | - | - |
| Yes | - | - | - | - | - |  | 0.9764 | <0.0001 | 2.6550 | 1.7271 | 4.0813 |  | - | - | - | - | - |
| MGMT | No (ref) | - | - | - | - | - |  |  |  |  |  |  |  | - | - | - | - | - |
| Yes | - | - | - | - | - |  | 0.4148 | 0.0739 | 1.5141 | 0.9607 | 2.3865 |  | - | - | - | - | - |
| MINT31 | No (ref) | - | - | - | - | - |  |  |  |  |  |  |  | - | - | - | - | - |
| Yes | - | - | - | - | - |  | 1.3916 | 0.0078 | 4.0212 | 1.4423 | 11.2115 |  | - | - | - | - | - |
| MLH1 | No (ref) | - | - | - | - | - |  |  |  |  |  |  |  | - | - | - | - | - |
| Yes | - | - | - | - | - |  | 0.8702 | 0.0582 | 2.3875 | 0.9702 | 5.8749 |  | - | - | - | - | - |
| NEUROG1 | No (ref) | - | - | - | - | - |  |  |  |  |  |  |  | - | - | - | - | - |
| Yes | - | - | - | - | - |  | 0.9069 | 0.0005 | 2.4766 | 1.4836 | 4.1340 |  | - | - | - | - | - |
| WIF1 | No (ref) | - | - | - | - | - |  |  |  |  |  |  |  | - | - | - | - | - |
| Yes | - | - | - | - | - |  | 0.7724 | 0.0012 | 2.1650 | 1.3570 | 3.4540 |  | - | - | - | - | - |
| MRS_10 | Low (ref) | - | - | - | - | - |  | - | - | - | - | - |  |  |  |  |  |  |
| Medium | - | - | - | - | - |  | - | - | - | - | - |  | 1.3474 | <0.0001 | 3.8475 | 2.7183 | 5.4457 |
| High | - | - | - | - | - |  | - | - | - | - | - |  | 1.8740 | <0.0001 | 6.5140 | 3.7661 | 11.2670 |

AUC: area under the curve; BMI: body mass index; CI: confidence interval; EF: environmental factor; MRS: methylation risk score; OR: odds ratio.

a A total of 428 CRC cases and 428 cancer-free controls were included in the development of the three models: EF-only, MRS_10, and combined EF and MRS_10 models. The regression coefficients, odds ratios and 95% CIs from each model were estimated and corrected based on the pooled multiple imputation datasets. For each model, the predicted probability of developing CRC can be determined from combination of predictor values; for example, for the EF-only model, the predicted probability of CRC risk is P = 1/[1 + exp - (0.0359 - 0.0898 × age + 0.1071 × gender - 0.8521 × BMI - 0.7768 × coarse grains + 0.5277 × fish stewed with brown sauce + 0.6488 × fried food + 1.1194 × occupational physical activity + 0.4067 × leftover + 0.3564 × pork + 0.3269 × smoking).
